# Gut Microbiota Modulation as a Therapeutic Strategy for Insomnia: A Systematic Review of Nutritional and Botanical Interventions

**DOI:** 10.3390/biom16070933

**Published:** 2026-06-23

**Authors:** Narada Vicharnnikornkij, Wanna Chaijaroenkul, Kesara Na Bangchang

**Affiliations:** 1 Graduate Program in Translational Biomedical Sciences and Innovation, Chulabhorn International College of Medicine, Thammasat University, Rangsit Campus, Pathumthani 12120, Thailand; narada.vicha@dome.tu.ac.th (N.V.); wn_ap39@yahoo.com (W.C.); 2 Center of Excellence in Pharmacology and Molecular Biology of Malaria and Cholangiocarcinoma, Thammasat University, Rangsit Campus, Pathumthani 12120, Thailand

**Keywords:** insomnia, microbiota–gut–brain axis, psychobiotics, prebiotics, probiotics, sleep architecture, circadian rhythm

## Abstract

*Background:* Insomnia and stress-related sleep disorders are increasingly recognized as systemic conditions linked to the microbiota–gut–brain axis (MGBA). With growing clinical interest in natural products that modulate the gut environment, this systematic review evaluates the efficacy and mechanisms of non-pharmacological interventions, specifically probiotics, prebiotics, dietary indices, and botanicals, in alleviating insomnia, restoring circadian rhythms, and modulating neurochemical markers. *Methods:* In strict accordance with PRISMA 2020 guidelines, we searched PubMed, ScienceDirect, Scopus, and The Cochrane Library for English language studies published from inception to March 31, 2026. Eligibility was restricted to studies with rigorously controlled designs, specifically randomized controlled trials (RCTs) and controlled in vivo animal studies. Interventions had to target the gut microbiota, with primary outcomes measuring sleep quality (subjective or objective) or sleep-related neurochemical markers. We excluded uncontrolled, single-arm, or observational designs; in vitro studies; non-original research; and studies involving subjects with severe medical or psychiatric comorbidities (e.g., cancer, ADHD, severe psychiatric disorders) to prevent confounding variables, though mild-to-moderate anxiety and depression were permitted. Risk of bias was assessed using the Cochrane RoB 2.0 and SYRCLE tools. Due to significant methodological heterogeneity, a narrative synthesis stratified by intervention and population was conducted. This review was not registered in PROSPERO. *Results:* A total of 56 studies (33 humans, 23 animals) met the inclusion criteria. Taxonomic nomenclature was updated to reflect 2020 reclassifications (e.g., *Lactiplantibacillus plantarum*). In human trials, interventions significantly improved subjective sleep metrics (PSQI, ISI). Recent additions demonstrated the efficacy of the Dietary Index for Gut Microbiota (DI-GM) and the improvement in N3 sleep latency by yeast mannan. Furthermore, whole-food patterns (e.g., the MIND diet) and Traditional Chinese Medicine (TCM) decoctions successfully enriched beneficial taxa, such as *Bacteroides coprophilus*, and increased short-chain fatty acid (SCFA) production. Animal models demonstrated that “psychobiotic” strains (*Bifidobacterium breve*, *Lacticaseibacillus paracasei*), prebiotics (GOS/PDX), and TCM formulas effectively restored GABA/5-HT profiles, lowered morning cortisol, and facilitated REM rebound in PCPA-induced models, while also consolidating non-rapid eye movement (NREM) sleep and downregulating clock genes (*Per1/Per2*). *Conclusions:* Psychobiotics, prebiotics, and botanicals represent a highly viable non-pharmacological strategy for treating insomnia. However, current evidence is constrained by a heavy reliance on subjective human questionnaires, short follow-up durations limiting insight into long-term stability, and a substantial translational gap between mechanistic rodent models and human clinical outcomes.

## 1. Introduction

Insomnia is a pervasive sleep disorder characterized by difficulty initiating or maintaining sleep, leading to significant distress and impaired daytime functioning. It affects approximately 10% to 30% of the global population and is frequently comorbid with psychiatric conditions such as anxiety and depression [1]. Current pharmacological interventions primarily target the central nervous system (CNS) through benzodiazepines and non-benzodiazepine receptor agonists, which are often limited by adverse effects, including cognitive impairment, daytime drowsiness, and risks of dependency [2]. This situation has prompted an urgent need for effective, safe non-pharmacological therapies that address the underlying physiological drivers of sleep dysregulation rather than merely inducing sedation.

Emerging research highlights the microbiota–gut–brain axis (MGBA) as a critical regulator of sleep physiology. The MGBA is a bidirectional communication network linking the enteric and central nervous systems, operating through neural (e.g., the vagus nerve), endocrine (the hypothalamic–pituitary–adrenal [HPA]-axis), and immune pathways, largely mediated by microbial metabolites [3]. Recent systematic reviews have substantiated this bidirectional relationship, indicating that gut dysbiosis, defined as an imbalance in microbial composition, correlates with sleep fragmentation and altered circadian rhythms [4,5]. Moreover, the role of orexin (hypocretin) in regulating wakefulness and arousal stability is gaining attention, as alterations in the gut microbiota may influence sleep architecture through neuroendocrine pathways converging on central arousal networks [6].

However, a distinct translational gap exists in understanding the precise mechanisms driving this relationship. In preclinical rodent models, specific microbial strains have been shown to modulate neurotransmitter synthesis directly, increasing hypothalamic levels of gamma-aminobutyric acid (GABA) and serotonin (5-hydroxytryptamine: 5-HT) while downregulating stress-induced cortisol [7,8]. Animal data suggest that short-chain fatty acids (SCFAs) produced by bacterial fermentation can cross the blood–brain barrier to influence sleep architecture [9] and improve symptoms of insomnia, induced anxiety-like behaviors, and cognitive dysfunction [10,11]. In contrast, human clinical observations have largely relied on associative data, linking reduced microbial diversity (e.g., depletion of *Faecalibacterium*) to chronic insomnia and systemic inflammation [12].

To leverage these pathways therapeutically, research has expanded beyond single-strain “psychobiotics,” a class of targeted probiotics (live microorganisms that confer a health benefit to the host), to encompass a broader array of non-pharmacological interventions [13]. These include prebiotics (non-digestible substrates selectively utilized by host microorganisms), whole-food dietary patterns, and complex botanical supplements. While numerous individual studies have explored interventions using *Lactobacillus* and *Bifidobacterium* strains, overall findings remain fragmented. To address this fragmentation, this systematic review aims to evaluate the current evidence on the efficacy of these interventions in alleviating insomnia, adopting a distinct translational framework that strictly separates human clinical outcomes from the neurochemical mechanisms observed in animal models. Human studies will be included to assess clinical efficacy, symptom management (via subjective and objective sleep parameters), and epidemiological dietary indices. In contrast, animal studies will be included strictly to demonstrate biological plausibility and delineate the potential neurochemical mechanisms, such as neurotransmitter regulation and HPA-axis suppression that may drive these therapeutic effects.

## 2. Methodology

### 2.1. Protocol and Registration

This systematic review was conducted following the Preferred Reporting Items for Systematic Reviews and Meta-Analyses (PRISMA) 2020 guidelines [14]. The protocol for this review was not registered in PROSPERO; however, a justification for this omission is provided in the discussion.

### 2.2. Search Strategy and Data Sources

A comprehensive literature search was performed in PubMed, ScienceDirect, Scopus, and The Cochrane Library for articles published from inception to March 31, 2026. To avoid selection bias toward mechanistic outcomes, specific mechanistic terms (e.g., “GABA,” “SCFA”) were excluded from the search string. The final Boolean search string used was: (“insomnia” OR “sleep initiation and maintenance disorders”) AND (“gastrointestinal microbiome” OR “gut microbiota” OR “probiotics” OR “prebiotics” OR “psychobiotics” OR “dietary fiber”). While no language restrictions were initially planned, only studies published in English were included in the final analysis to ensure clarity and comprehensibility.

### 2.3. Eligibility Criteria

To ensure a rigorous selection process, studies were evaluated against specific inclusion and exclusion criteria. Inclusion criteria were as follows: (i) Strictly controlled study designs, limited to randomized controlled trials (RCTs) or controlled in vivo animal studies, (ii) Interventions targeting the gut microbiota (e.g., probiotics, prebiotics, dietary indices, botanicals), and (iii) Primary outcomes measuring sleep quality (subjective or objective) or sleep-related neurochemical markers. During the review process, we recognized the value of certain human observational and cross-sectional studies. Consequently, a limited number of these studies were included to provide contextual evidence, particularly regarding dietary index analyses. The inclusion criteria were thus adapted to allow for a broader understanding of the relationship between diet and sleep. Exclusion criteria included: (i) Uncontrolled, single-arm, or observational study designs not meeting the relevance threshold, (ii) Publications in a non-English language that did not contribute significant insights, (iii) Non-original research, including narrative reviews, systematic reviews, editorials, case reports, and conference abstracts, (iv) In vitro studies, and (v) Studies involving subjects with severe medical or psychiatric comorbidities to prevent confounding variables. Specifically excluded comorbidities were any oncological diagnoses (cancer), Attention Deficit Hyperactivity Disorder (ADHD), and severe psychiatric disorders (although mild-to-moderate anxiety and depression were permitted). During the review process, we recognized the value of certain human observational and cross-sectional studies. Consequently, a limited number of these studies were included to provide contextual evidence, particularly regarding dietary index analyses. The inclusion criteria were thus adapted to allow for a broader understanding of the relationship between diet and sleep.

### 2.4. Analytical Framework and Risk of Bias

To prevent the inappropriate extrapolation of animal mechanisms to human clinical outcomes, our analysis was strictly stratified. The Risk of Bias was assessed using the RoB2 tool for human RCTs and the SYRCLE tool for animal studies.

### 2.5. Evidence Level Assignment

Interventions were categorized into evidence levels such as “High,” “Moderate-High,” etc. The criteria for these classifications were based on a combination of factors, including: (i) Study design quality (e.g., RCTs rated higher than observational studies); (ii) consistency of findings across studies; (iii) directness of evidence regarding the effect on sleep quality; and (iv) precision of results (e.g., confidence intervals and effect sizes). While a formal framework such as GRADE was not used, we employed a semi-structured approach to ensure evidence levels were assigned systematically. In cases of conflicting findings, we conducted a qualitative synthesis to assess the strength of the evidence. We considered factors such as sample size, methodological rigor, and potential biases in the studies. Conflicts were discussed in detail, and we provided a narrative explanation for the assigned evidence level to ensure transparency in our assessment.

## 3. Results

### 3.1. Study Selection and Characteristics

**Figure 1:**
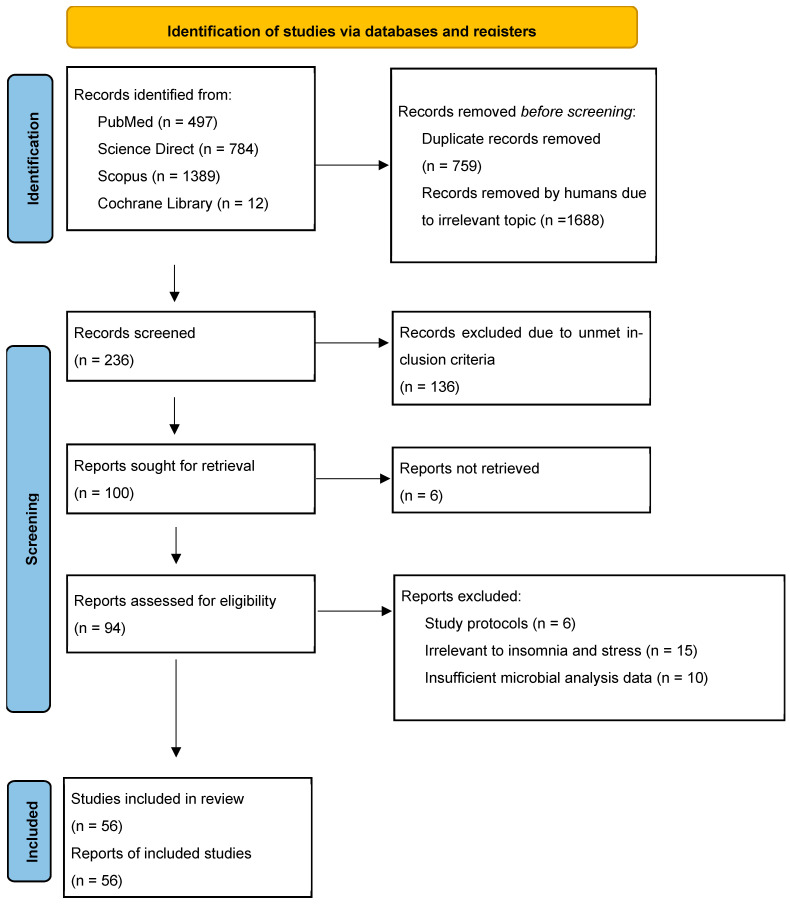
Flow chart of the systematic review.

The database search identified 2682 records. Following the removal of duplicates and title/abstract screening, 236 articles were assessed for eligibility. Ultimately, 56 studies were included in the final analysis (33 human clinical studies and 23 animal models) (Figure 1). The included studies exhibited significant heterogeneity regarding intervention type, participant demographics, and dosage, which ranged widely from 10^8^ to 10^11^ CFU. Methodologies used in clinical and in vivo studies are summarized in Table 1, and key parameters for both study types are summarized in Table 2. A summary of all clinical and animal studies is presented in Supplementary Tables S1 and S2, respectively.

*Human clinical trials:* Human studies were evaluated strictly for clinical efficacy, primarily utilizing subjective sleep questionnaires such as the Pittsburgh Sleep Quality Index [PSQI] and Insomnia Severity Index [ISI]. However, the current body of evidence is constrained by reliance on subjective metrics, which are prone to recall and placebo bias, as well as by 16S rRNA resolution limits and short follow-up durations. Interventions across these trials broadly spanned probiotics, prebiotics, broad dietary indices, and traditional botanicals.

*Animal models:* Animal studies primarily utilizing rodent models of p-chlorophenylalanine (PCPA)-induced insomnia or acute sleep disruption were analyzed to establish biological plausibility. These mechanisms demonstrate preclinical potential rather than definitive human causation, focusing on how interventions modulate secondary bile acids, lower morning cortisol, and regulate central neurotransmitters via gut-derived metabolites.

### 3.2. Stratified Analysis of Probiotic Interventions (Human RCTs)

To address variability in outcomes, psychobiotic interventions were stratified by bacterial genus and strain to identify specific patterns of efficacy.

*Subgroup A—Lactobacillus strains*: Evidence suggests high strain specificity in subjective sleep quality. *Lactobacillus helveticus* CCFM1320 [15], *Lacticaseibacillus paracasei* CP2305 (Table S1), *Lacticaseibacillus paracasei* 207-27 [17], and *Lacticaseibacillus paracasei* K56 [21] demonstrated consistently strong efficacy in reducing PSQI scores and sleep latency in both healthy adults and elderly cohorts [16]. In contrast, *Lactobacillus rhamnosus* Lpc-37 failed to alter stress-related sleep measures in healthy subjects who undergo stress [36]. While *Lacticaseibacillus paracasei* 207-27 improved subjective quality in stressed populations, it failed to demonstrate significant changes in objective actigraphy parameters (e.g., total sleep time) compared to the placebo [17]. This divergence suggests that the efficacy of Lactobacillus strains may depend on baseline stress levels, highlighting the need for tailored probiotic interventions based on individual stress profiles.

*Subgroup B—Bifidobacterium strains:* Interventions using Bifidobacterium species showed a trend toward improving sleep efficiency and architecture, rather than just subjective sleep scores. *Bifidobacterium breve* 207-1 was associated with an increased sleep efficiency (Table S1), while *Bifidobacterium animalis* subsp. lactis BB-12, particularly when combined in synbiotic formulations, significantly mitigated cortisol awakening responses within 4 weeks [22]. The ability of Bifidobacterium strains to influence both subjective and objective sleep measures underscores their potential as effective psychobiotics in insomnia management.

*Subgroup C—multi-strain and synbiotic formulations:* Studies using multi-strain formulations generally reported a faster onset of action than those using single-strain formulations. However, a “responder” effect was a critical variable; the efficacy of these interventions often correlated with the baseline abundance of *Faecalibacterium prausnitzii*, suggesting that a host’s pre-existing gut ecology gates the therapeutic success of multi-strain supplements [35]. This emphasizes the importance of personalized approaches in probiotic therapy, accounting for the individual microbiome landscape.

### 3.3. Impact of Dosage, Duration, and Population Heterogeneity

A comparative analysis of the included RCTs reveals that doage thresholds and population characteristics largely drive variability in clinical outcomes.

*Dosage and duration:* Interventions demonstrating significant improvements in sleep efficiency typically employed dosages spanning 10^8^ to 10^11^ CFU/day with intervention durations of at least 8 weeks. Studies with shorter durations (<4 weeks) or lower dosages frequently reported non-significant results for objective sleep parameters, suggesting a time-dependent requirement for colonization or metabolic modulation.

*Population specificity:* There is a notable “stress-dependent” efficacy. Probiotics consistently produced larger effect sizes in populations with high baseline physiological stress (e.g., medical students, shift workers) compared to healthy, low-stress controls. This suggests that psychobiotics may primarily function by restoring homeostasis in dysregulated HPA axis conditions rather than enhancing sleep in healthy homeostatic states.

### 3.4. Effects of Prebiotic, Botanical, and Dietary Interventions (Human)

Non-probiotic interventions focused on metabolic modulation through dietary fibers, comprehensive diet patterns, and fermented botanical extracts.

*Prebiotics:* Prebiotic interventions utilizing galactooligosaccharides and polydextrose (GOS/PDX) were associated with improved sleep continuity and specifically enhanced rapid eye movement sleep (REM) duration and sleep quality in adults [26,37,38]. Persistent results were obtained in neonatal and infant subjects, with increasing daytime sleep duration and shorter sleep latency [39,40]. A recent double-blind RCT demonstrated that ingestion of yeast mannan significantly lengthened the total sleep time, shortened N3 sleep latency on electroencephalogram (EEG), and improved bowel habits in healthy adults, with effects mediated by increases in beneficial gut metabolites [18,23].

*Dietary Indices:* Expanding beyond isolated supplements, the Dietary Index for Gut Microbiota (DI-GM) was validated in a cross-sectional study of adults [39]. Higher DI-GM scores were strongly correlated with improved sleep quality, lower depression/anxiety, and reduced intestinal and systemic inflammation, underscoring the macro-level impact of gut-friendly diets [41]. Similarly, whole-food interventions, such as the Mediterranean-DASH Intervention for Neurodegenerative Delay (MIND) diet [42], high dietary fibers [43,44], and farm-based cookery [45], have been shown to increase microbial diversity and SCFA production. Additionally, some specific dietary patterns [46,47,48] and vitamin levels [49] have been reported to influence sleep quality and mental well-being, related to the diversity and balance of the gut microbiome profile. These findings suggest that dietary patterns significantly influence not only gut health but also sleep quality and mental well-being.

*Botanicals and TCM*: Botanical interventions frequently employ fermentation or tradi-tional decoction methods. Flavonoid-rich extracts from blackcurrant [50] and ginger [51] effectively modulated gut microbiome composition and improved mental well-being. Furthermore, an interventional trial demonstrated that TCM formulas (CSQBD and STYHCD) significantly improved ISI and PSQI scores among patients with insomnia [24]. These clinical benefits were directly linked to the enrichment of specific commensals, such as *Bacteroides coprophilus*, and the modulation of anti-inflammatory cytokines. Other traditional medicines, such as Japanese Kamikihito kampo, also demonstrated interventions that revealed the potential of traditional medicine to improve psychological stress and other aspects of mental well-being [52].

### 3.5. Preclinical Findings (Animal Models)

Animal studies have provided objective verification of changes in sleep architecture and precise neurochemical mechanisms that are currently difficult to quantify in humans.

*NREM and REM rebound:* Administration of Bifidobacterium subspecies and Bifidobacterium breve significantly increased NREM sleep duration and enhanced delta power (an indicator of sleep depth) (Table S2). Moreover, some probiotic strains, such as high-lactic-acid-producing bacteria, can alleviate stress and promote the restoration of gut microbiome composition disrupted by stress and sleep deprivation (Table S2). Similarly, a robust prebiotic diet was shown to alter the fecal microbiome and facilitate the recovery of critical sleep architecture (REM rebound) following acute sleep disruption in rats. Similar results of GOS/PDX prebiotic intervention capable of improving REM sleep, sleep patterns, and providing anxiolytic properties were persistent in most animal trials [19,25,26,53,54].

*Restoration of neurotransmitters* via *botanicals and fermentation:* Recent models of PCPA-induced insomnia have strongly validated the efficacy of complex botanicals. Suanzaoren tang was shown to directly mitigate insomnia via integrated metabolomics [29], while Lily-Ziziphi Spinosae Semen and Banxia-Yiyiren decoctions were shown to restore normal sleep–wake cycles by regulating the gut microbiota and rebalancing brain GABA/glutamate ratios [30,31]. Botanical extracts have been studied for their sleep-promoting and stress-soothing properties [32,33,55], but GLAA extract and eucalyptus essential oil were found to promote sleep, decrease the expression of wakefulness genes, and modulate the levels of sleep-promoting neurotransmitters [32,56]. Given neurotransmitters, as a supplement, have been found to help restore circadian rhythm [34] and alleviate sleep-deprived or chronic stress-induced anxiety and depression behaviors [57,58].

*Anxiety-linked sleep disruption:* In murine models of stress-induced insomnia, *Limosilactobacillus reuteri* WLR01 specifically reversed social-defeat stress behaviors and restored normal sleep–wake cycles, an effect not observed with other Lactobacillus strains in the same study [59].

### 3.6. Biological Mechanisms (Preclinical vs. Clinical)

This review identified four primary biological mechanisms by which the interventions influenced sleep. It is critical to note which biomarkers were identified in human serum and which were inferred from rodent brain tissue.

*HPA-axis modulation (humans and animals):* The HPA-axis is a central component of the body’s stress response system. Its regulation is crucial for maintaining homeostasis and modulating sleep patterns. The HPA-axis involves a complex feedback loop that starts with the hypothalamus releasing corticotropin-releasing hormone (CRH). This hormone stimulates the pituitary gland to produce adrenocorticotropic hormone (ACTH), which in turn prompts the adrenal glands to release cortisol. Multiple RCTs have shown significant reductions in morning serum cortisol levels following probiotic administration. For example, one study found that participants consuming a specific probiotic blend exhibited lower cortisol levels and improved sleep quality. Salivary cortisol measurements in these studies serve as a non-invasive biomarker for stress and sleep quality, providing a direct link between gut microbiota modulation and HPA-axis activity. Animal models have corroborated these findings, showing that probiotic strains such as *Lactobacillus* spp. can significantly reduce stress-related hormones, indicating a similar regulatory mechanism (Table S1) [17,25].

*Neurotransmitter regulation (animals only):* Gut bacteria synthesize various neuroactive compounds, such as GABA and serotonin. These neurotransmitters are vital for promoting relaxation and sleep onset. Invasive studies in rodent models have directly quantified levels of these neurotransmitters in brain tissue. For instance, interventions involving Lactobacillus species significantly elevated GABA and 5-HT levels in the hypothalamus and frontal cortex. Research has shown that these elevations correlate with improved sleep quality and reduced anxiety, highlighting the role of gut microbiota in influencing central nervous system function and behavior (Table S1) [21,33]. While these findings are currently limited to animal studies, they suggest a promising avenue for future research to explore similar effects in humans. Understanding how gut microbiota affects neurotransmitter synthesis could lead to novel treatments for sleep disorders.

*Circadian gene expression (animals only):* The regulation of circadian rhythms is another critical mechanism through which gut microbiota can influence sleep. Circadian rhythms are controlled by core clock genes, such as *Per1* and *Per2*, which regulate the sleep–wake cycle. RT-qPCR analyses in rodent models have demonstrated that specific botanical extracts, including those from *Bacopa monnieri*, can downregulate the expression of these core circadian genes (Table S2) [34]. This downregulation is significant, especially in the context of sleep deprivation, where these genes are typically upregulated. Normalizing their expression may help restore healthy sleep patterns. These findings suggest that botanical interventions could be used not only to improve sleep but also to help regulate circadian rhythms, offering a holistic approach to managing sleep disorders.

*SCFAs and Bile Acids production (humans and animals):* SCFAs and bile acids produced by gut microbiota have been identified as important metabolites affecting sleep. SCFAs, such as butyrate and propionate, are produced during the fermentation of dietary fibers by gut bacteria. They serve as signaling molecules that can influence various physiological processes, including sleep. Metabolomic profiling has shown that increased levels of butyrate and propionate are associated with improved sleep consolidation in both human and animal studies. Elevated concentrations of these SCFAs were measured in fecal and serum samples from participants undergoing probiotic or prebiotic interventions. Additionally, the modulation of secondary bile acids has been observed, further supporting the gut–brain axis’s role in sleep regulation [10,26,33]. The correlation between SCFA levels and NREM sleep consolidation suggests that these metabolites could be targeted in therapeutic interventions to improve sleep quality.

*HPA axis modulation (humans and animals):* The most frequently validated mechanism across both species was the attenuation of the stress response. Multiple RCTs and animal models confirmed significant reductions in morning serum and salivary cortisol, ACTH, and CRH following probiotic administration [2,3,17,25]. The HPA-axis is a central component of the body’s stress response system. Its regulation is crucial for maintaining homeostasis and modulating sleep patterns. The HPA-axis involves a complex feedback loop that starts with the hypothalamus releasing CRH. This hormone stimulates the pituitary gland to produce ACTH, which in turn prompts the adrenal glands to release cortisol. Multiple RCTs have shown significant reductions in morning serum cortisol levels following probiotic administration. For example, one study found that participants consuming a specific probiotic blend exhibited lower cortisol levels and improved sleep quality. Salivary cortisol measurements in these studies serve as a non-invasive biomarker for stress and sleep quality, providing a direct link between gut microbiota modulation and HPA-axis activity. Animal models have corroborated these findings, showing that probiotic strains such as Lactobacillus spp. can significantly reduce stress-related hormones, indicating a similar regulatory mechanism [2,3,17,25].

Metabolomic profiling identified elevated concentrations of butyrate and propionate, alongside the modulation of secondary bile acids, in fecal and serum samples of treated groups [10,26,33]. These increases correlated positively with NREM sleep consolidation, supporting the role of SCFAs as key signaling molecules in the gut–brain axis.

### 3.7. Risk of Bias Assessment

The individual risk-of-bias assessments are detailed in Supplementary Table S3A,B (humans) and S4 (animals). Among the 33 human clinical trials, 16 were classified as ‘Low Risk’ across all RoB 2.0 domains. ‘Some concerns’ were frequently noted in the ‘randomization process’ domain, particularly in older studies where the method of allocation concealment was not explicitly described. Five studies were flagged as ‘High risk’ due to missing outcome data and an inadequate statistical correction (intention-to-treat analysis). Among the 23 animal studies assessed via SYRCLE, the reporting quality varied. While ‘baseline characteristics’ and ‘selective reporting’ were generally low-risk, the domains of ‘random housing’ and ‘blinding of caregivers’ were frequently assessed as ‘Unclear risk’ due to insufficient reporting details in the methodology sections. This lack of blinding reporting may introduce performance bias into the preclinical data. Moving forward, future research must utilize functional metagenomics, objective polysomnography (PSG/EEG), functional neuroimaging, and Mendelian randomization to validate causal determinants and establish standardized therapeutic protocols. Table 3 presents the strength of evidence for various interventions, and Table 4 presents the reliability of the tools used to measure sleep and microbiome changes. Table 5 provides a comparative summary of intervention patterns.

### 3.8. The Role of Orexin in Sleep Regulation and Gut Microbiota Interactions

Orexin (also known as hypocretin) is a neuropeptide produced in the hypothalamus that plays a critical role in regulating wakefulness, arousal stability, and the overall sleep–wake cycle. Recent research highlights the intricate relationship between gut microbiota and orexinergic pathways, suggesting that alterations in gut microbial composition may significantly influence sleep architecture through neuroendocrine and inflammatory pathways that converge on central arousal networks [62]. Emerging evidence indicates that gut microbiota can modulate the levels of orexin and its receptors, thereby impacting wakefulness and sleep quality. Certain gut-derived metabolites, such as SCFAs, may influence the hypothalamic release of orexin, thereby promoting arousal and alertness. This connection underscores the potential for gut microbiota to affect not only digestive health but also neurochemical pathways integral to sleep regulation. Alterations in gut microbiota can elicit neuroendocrine responses that affect the HPA-axis, which plays a pivotal role in stress regulation and sleep patterns. Dysregulation of the HPA-axis can lead to increased cortisol levels, which are associated with heightened arousal and disrupted sleep. By influencing the HPA axis, alterations in the gut microbiota may subsequently affect orexin signaling, leading to changes in sleep architecture characterized by reduced REM sleep and increased wakefulness. Chronic inflammation, often resulting from dysbiosis (an imbalance in gut microbiota), can trigger neuroinflammatory processes that further disrupt sleep. Inflammatory cytokines can directly influence orexin neurons, potentially impairing their function and altering sleep patterns. Increased levels of pro-inflammatory cytokines may inhibit orexin release, leading to decreased arousal stability and increased susceptibility to sleep disturbances. The interplay between gut microbiota, orexin, and sleep regulation presents promising avenues for therapeutic interventions. Targeting gut microbiota through dietary modifications, prebiotics, or probiotics may enhance orexin signaling and improve sleep outcomes. Such approaches could be particularly beneficial in populations experiencing insomnia or sleep disorders linked to stress and inflammation.

## 4. Discussion

In this systematic review, we aimed to explore the relationship between gut microbiota modulation and sleep quality, focusing on dietary interventions. Our findings indicate significant interest in this research area; however, substantial heterogeneity among studies complicates our ability to draw definitive conclusions.

### 4.1. Heterogeneity in Evidence

The variability in study designs, populations, and outcome measures necessitates careful interpretation of the results. While narrative synthesis was justified due to this heterogeneity, a more detailed exploration of the factors contributing to this variability would strengthen our analysis. Differences in intervention types, dosages, and assessment tools were significant barriers to conducting a quantitative meta-analysis.

### 4.2. Consideration of Subgroup Meta-Analysis

We considered the potential for subgroup meta-analyses, particularly for interventions like probiotics assessed with the PSQI. However, the variability in methodologies and outcome reporting across studies precluded the feasibility of quantitative pooling. Discrepancies in reporting made it challenging to establish a uniform approach for synthesis.

### 4.3. Importance of Standardization

The potential to synthesize specific subsets, such as probiotic RCTs using PSQI, underscores the need for more standardized methodologies in future research. Standardization of outcome measures and study designs will facilitate more robust meta-analytic evaluations, ultimately strengthening the evidence base in this field.

### 4.4. Chronic Gut-Mediated Inflammatory Conditions

A comprehensive discussion of chronic gut-mediated inflammatory conditions, such as celiac disease, is warranted, as these conditions can model microbiota-gut–brain axis dysfunction that contributes to sleep disturbances. Emerging evidence suggests that celiac disease is associated with neurological and neuropsychiatric symptoms, systemic inflammation, altered gut permeability, dysbiosis, and neuroimmune activation. These mechanisms may overlap with pathways implicated in insomnia and stress-related sleep disorders, including HPA-axis dysregulation, cytokine imbalance, and neuroinflammation.

### 4.5. Synthesis of Evidence and Strain Specificity

This review synthesizes data from 33 clinical studies and 23 animal models to evaluate the efficacy of gut microbiota modulation in insomnia treatment. While psychobiotics offer a promising therapeutic avenue, efficacy is not uniform across the genus but is highly strain-specific. For instance, specific strains like *Lactobacillus helveticus* CCFM1320 and *Lacticaseibacillus paracasei* CP2305 significantly improve PSQI and sleep efficiency, whereas Lactobacillus rhamnosus JB-1 lacks clinical efficacy, emphasizing the risk of generalizing findings at the species or genus level. Furthermore, our synthesis suggests a dose-dependent threshold, with consistent improvements in sleep architecture observed in trials using dosages exceeding 10^9^ CFU/day for at least 8 weeks.

### 4.6. Ecological Predictors vs. Interventional Outcomes

A critical distinction must be drawn between the effects of administered interventions and the predictive value of the native gut ecosystem. Several studies indicated that the abundance of specific commensal taxa, particularly *Faecalibacterium prausnitzii* and Bifidobacterium species, correlates with treatment efficacy. However, these observations are largely derived from secondary analyses rather than direct interventional manipulation of these specific taxa.

### 4.7. Mechanistic Pathways: Distinguishing Animal Causal Models from Human Correlations

The mechanistic understanding of the gut–brain-sleep axis relies on distinct lines of evidence. Causal evidence from animal models demonstrates links between bacterial administration and sleep physiology, while human trials offer correlative evidence, lacking direct assessments of central nervous system neurotransmitter levels. Thus, while B. longum administration correlates with reduced stress markers in humans, the direct modulation of brain GABA/5-HT receptors remains inferred from animal studies.

### 4.8. The Role of Stress, Prebiotics, Botanicals, and Holistic Diets

Our analysis reveals a shift towards complex, multi-target interventions. Trials using prebiotics and broad dietary patterns aim to stimulate native flora to produce systemic anti-inflammatory markers and SCFAs. Notably, interventional trials in high-stress populations yield larger effect sizes, suggesting that holistic microbiota modulation primarily mitigates stress-induced hyperactivation of the HPA axis rather than directly inducing sedation.

### 4.9. Methodological Quality and Risk of Bias

A significant limitation of the current evidence base is the methodological heterogeneity and risk of bias within the included studies. Concerns regarding randomization processes and allocation concealment in several older RCTs may inflate positive effect sizes. Moreover, the high variability in dosing regimens complicates comparisons and dose–response analyses.

### 4.10. Limitations and Future Directions

A major limitation of the existing literature is the reliance on subjective clinical data, particularly self-reported questionnaires like the PSQI and ISI. The heterogeneity of reviewed studies poses challenges for clinical standardization, with variations in probiotic delivery matrices and dosages limiting the strength of meta-analytic conclusions. To overcome these limitations, future research must transition to objective sleep measurements, such as polysomnography (PSG) or actigraphy, to definitively demonstrate the efficacy of microbiota-gut–brain axis-targeted interventions.

In conclusion, modulating the gut microbiota through broad dietary indices, targeted prebiotics, and specific botanical formulations represents a promising approach to insomnia management. While animal models robustly establish the biological plausibility of this neurochemical modulation, translating these findings into standardized human clinical protocols requires rigorously designed, objective-based trials. Bridging the gap between preclinical mechanisms and clinical efficacy is essential for evolving gut microbiota modulation into a standardized therapeutic strategy for chronic insomnia.

## Figures and Tables

**Table 1:** Summary of methodologies applied in clinical and in vivo studies.

Methodologies	Clinical (Human) Studies	In Vivo (Animal) Studies
Primary parameters	Symptom management, clinical efficacy, subjective sleep quality	Biological plausibility, neurochemical mechanisms, objective verification
Sleep data	Subjective questionnaires (PSQI, ISI), Actigraphy, EEG/Polysomnography (e.g., N3 latency)	Objective sleep architecture (EEG), NREM/REM rebound, delta power
Microbiome	Fecal 16S rRNA, Specific commensal enrichment, Dietary Indices (DI-GM)	Fecal microbiome alteration, Integrated metabolomics
Biochemistry and Mechanisms	Serum/Saliva cortisol, ACTH, CRH, anti-inflammatory cytokines, Fecal/Serum SCFAs (butyrate, propionate), secondary bile acids	Brain tissue neurotransmitters (GABA/Glutamate ratios, 5-HT), clock genes (*Per1*, *Per2* via RT-qPCR), secondary bile acids
Study Models and Controls	Placebo-matched, baseline stress stratification (high-stress vs. healthy populations)	PCPA-induced insomnia, acute sleep disruption, social-defeat stress, vehicle-control

**Table 2:** Summary of key parameters applied in clinical and in vivo studies.

Parameter	Systematic Finding	Key References
Primary sleep outcomes	Subjective improvements (PSQI, ISI scores); objective EEG improvements (shortened N3 latency, total sleep time); preclinical NREM/REM rebound and enhanced delta power.	[15,16,17,18,19,20]
Secondary outcomes	Attenuation of the HPA axis (lowered morning cortisol, ACTH, and CRH); reduction in depression, anxiety, and systemic/anti-inflammatory cytokines.	[17,21,22,23,24,25]
Microbial shifts and Indices	Efficacy gated by baseline *Faecalibacterium prausnitzii*; enrichment of *B. breve*, *Lactobacillus* spp. (*CCFM1320*, *CP2305*), and *Bacteroides coprophilus*; validated by high DI-GM scores.	[15,17,23,24,26,27,28], Table S1
Validated mechanisms	Restoration of brain GABA/glutamate ratios and 5-HT; Downregulation of clock genes (*Per1*, *Per2*); increased SCFAs (butyrate, propionate) and secondary bile acids.	[26,29,30,31,32,33,34]
Optimal dosage and duration	>8 weeks was required for significant changes in objective sleep parameters; dosage thresholds of 10^8^ to 10^11^ CFU/day; higher efficacy in high-stress populations.	[15,17,21,22,35]

**Table 3:** Categorization of interventions and evidence levels.

Intervention	Study Design	Evidence Level	Criteria for Classification	Comments on Conflicting Findings
Probiotics	RCTs	High	Strong evidence from multiple RCTs showing consistent improvement in sleep quality [15,17,21,36].	Conflicting results noted; analyzed differences in strains and dosages.
Prebiotics	RCTs	Moderate-High	Some RCTs show positive effects, but variability in outcomes across studies [18,25,35,40,51,52,54].	Variability attributed to differences in population characteristics and intervention duration.
Dietary Indices	Observational	Moderate	Correlational evidence from observational studies; supportive but not causal [23,42,47].	Conflicting findings exist; discussed potential confounding factors such as lifestyle and dietary habits.
Botanicals	RCTs & Observational	Moderate	Mixed evidence from both RCTs and observational studies; some positive effects noted	Conflicts resolved by emphasizing the need for further RCTs to confirm results.
Gut Microbiota Modulation	Animal Studies	High	Strong mechanistic evidence from animal studies; direct links to neurotransmitter activity	While evidence is robust in animals, caution is advised when extrapolating to humans.

Notes: Evidence Level Definitions: High = Strong, consistent evidence from multiple well-conducted RCTs, Moderate-High = Evidence from RCTs with some inconsistencies or limited scope, Moderate = Correlational or observational studies with some supportive findings but lacking direct causal links. Each intervention’s comments provide insights into how conflicts were analyzed, emphasizing the importance of understanding study context and potential confounding variables.

**Table 4:** Reliability of tools used to measure sleep and microbiome changes.

Tool/Method	Reliability Score	Rationale
EEG/Polysomnography (PSG)	Very High	The objective “Gold Standard.” Provides real-time data on sleep architecture (N3 latency, NREM/REM rebound) and depth (Delta power). Highlighted as a necessary standard for future human trials to overcome subjective bias.
RT-qPCR and Brain Tissue Analysis (Animal)	Very High	Directly quantifies neuroactive compounds (GABA, 5-HT, Glutamate ratios) and core clock gene expression (*Per1*, *Per2*) to establish precise biological plausibility currently unobtainable in humans.
Subjective Questionnaires (PSQI & ISI)	Moderate	Widely used for assessing symptom management and clinical efficacy in humans, but constrained by a high susceptibility to recall and placebo biases.
16S rRNA Sequencing	Moderate	Standardized for taxonomic identification of specific commensals, but constrained by species/strain resolution limits. The review explicitly notes that future research must shift to functional metagenomics to capture actual metabolic activity.
Actigraphy (Humans)	Moderate	Provides non-invasive tracking of sleep–wake parameters (e.g., total sleep time), but less precise than PSG/EEG. Interventions sometimes fail to show objective changes on actigraphy despite subjective improvements.
Serum and Salivary HPA Biomarkers	High *(Protocol Dependent)*	Reliable indicators of HPA axis attenuation (cortisol, ACTH, CRH) and baseline stress stratification, provided morning sampling protocols are strictly controlled to account for circadian variability.
Functional Neuroimaging and Mendelian Randomization	Future Gold Standard	Identified in the review as the necessary next-generation methodologies required to definitively validate causal determinants between gut interventions and central nervous system activity.

**Table 5:** Comparative summary of intervention patterns.

Human Studies					
Intervention Type	Specific Strain/Compound	Study Subject	Duration	Key Outcome	Ref.
Probiotic	*Lacticaseibacillus paracasei* CP2305	Athletes	12 weeks	Reduced anxiety and fatigue; increased *Faecalibacterium*	(Table S1)
Probiotic	*Bifidobacteriusm breve* 207-1 (High dose)	Healthy	4 weeks	Improved PSQI score; increased brain GABA	(Table S1)
Probiotic	*Lactobacillus helveticus* CCFM1320	Insomnia	4 weeks	Improved PSQI; increased serum SAMe	[15]
Probiotic	*Lacticaseibacillus paracasei* K56	Students	4 weeks	Reduced Insomnia Severity Index (ISI) and stress	[21]
Synbiotic	Probiotic + Prebiotic Ice Cream	Military	15 days	Improved PSQI; reduced tenseness under extreme stress	[22]
Prebiotic	Fiber-enriched Kombucha	Healthy	4 weeks	Increased *Bifidobacterium*; reduced total cholesterol	[27]
Dietary Index:	DI-GM	Healthy	Cross sectional	Reduced depression/anxiety symptoms; improved sleep and reduced inflammation	[23]
Prebiotic	Yeast Mannan	Healthy	RCT	Lengthened total time in bed; shortened N3 sleep latency	[18]
Botanical (TCM):	CSQBD and STYHCD	Insomnia	RCT	Improved ISI/PSQI; enriched Bacteroides coprophilus	[24]
Animal Studies					
Intervention Type	Specific Strain/Compound	Animals	Duration	Key Outcome	Ref
Probiotic	*Lactobacillus reuteri* WLR01	Mice (sleep deprived)	2 weeks	Reduced anxiety; restored cognitive function	[59]
Prebiotic	GOS/PDX	Rats (stress/CDR)	Early life/Chronic	Faster CBT realignment; REM rebound	[26,53]
Natural product	High-GABA fermented milk	Mice	Single/short term	Reduced sleep latency; increased sleep duration	(Table S2)
Natural product	*Moringa oleifera* (fermented)	Mice	1 week	Increased Brain GABA; improved Glutamate/GABA ratio	[33]
Natural product	BSSC (Crude Extract)	Mice (sleep deprived)	2 weeks	Downregulated *Per1*/*Per2*; reduced eosinophils	(Table S2)
Prebiotic	Prebiotic Diet	Rats (Sleep deprived)	Short term	Altered fecal microbiome; improved sleep recovery/REM rebound	[19]
Botanical (TCM):	Suanzaoren tang/Banxia-Yiyiren	Mice/Rats (PCPA-induced)	Short term	Alleviated insomnia/anxiety; restored neurotransmitters via gut metabolites	[29,31]
Natural Product	Fermented germinated grain/Xizang dairy Lactobacillales	Mice	Short term	Restored neurotransmitter profiles; improved sleep quality	[60,61]

## Data Availability

All data generated or analyzed during this study are included in the published article.

## Supplementary Materials

The following supporting information can be downloaded at: https://www.mdpi.com/article/10.3390/biom16070933/s1, Supplementary Table S1: Summary of clinical studies involving the roles of nutritional and herbal interventions on gut microbiota modulation as a therapeutic strategy for the treatment of insomnia. Supplementary Table S2: Summary of in vivo studies involving the roles of nutritional and herbal interventions on gut microbiota modulation as a therapeutic strategy for the treatment of insomnia. Supplementary Table S3A: Human intervention studies (28 Studies). Supplementary Table S3B: Human observational/cross-sectional studies (5 Studies). Supplementary Table S4: Animal (In Vivo) Studies (23 Studies). Table S5: PRISMA 2020 checklist; Table S6: PRISMA 2020 abstract checklist.

## Abbreviations

The following abbreviations are used in this manuscript:

ACTH	Adrenocorticotropic Hormone
AGP	Alpha-1 Acid Glycoprotein
AIS	Athens Insomnia Scale
aOR	Adjusted Odds Ratio
BDNF	Brain-Derived Neurotrophic Factor
BDI II	Beck Depression Inventory-second edition
CFS	Chadler Fatigue Scale
CSHQ	Children’s Sleep Habits Questionnaire
CRH	Corticotropin-releasing hormone
CBT	Core Body Temperature
CFU	Colony-Forming Unit
CSQBD	Traditional Chinese Medicine Decoction
CRP	C-Reactive Protein
DASS	Depression Anxiety Stress Scale
DP	Diet Pattern
DP-I	Diet Pattern I (high loading of bean products, coarse grain, nuts, fruits, mushrooms, and potatoes)
DP-II	Diet Pattern II (Dark &light vegetables, red meat, poultry, rice, and liver)
DP-III	Diet Pattern III (Congee, dessert, eggs, and stuffing food)
DP-IV	Diet Pattern IV (Fried food, julep, and processed meat product)
DI-GM	Dietary Index for Gut Microbiota
EMG	Electromyography
EEG	Electroencephalography
ESS	Epworth Sleepiness Scale
EPM	Elevated Plus Maze
ELISA	Enzyme Link Immune Assay
FOSQ	Functional Outcome of Sleep Questionnaire
FCT-C	Functional Assessment of Cancer
FST	Forced Swim Test
GCMS	Gas Chromatography Mass Spectrometry
GSRS	Gastrointestinal Symptoms Rating Scale
GABA	Gamma-Aminobutyric Acid
GMBs	Gut–Brain Modules
Glu	Glutamate
HAM-D	Hamilton Depression Rating Scale
EQ-5D-3L	Health-Related Quality Of Life EuroQol-5 Dimension-5 Level
HPLC	High Performance Liquid Chromatography
HADS	Hospital Anxiety and Depression Scale
5-HT	5-Hydroxytryptamine
5-HTP	5-Hydroxytryptophan
5-HIAA	5-Hydroxyindoleacetic Acid
HPA	Hypothalamic–Pituitary–Adrenal (Axis)
IPAQ	International Physical Activity Questionnaire
ISI	Insomnia Severity Index
IL-6	Interleukin-6
IGSQ	Infant Gastrointestinal Symptom Questionnaire
IL-1β	Interleukin I-beta
IFN-α	Interferon-gamma
JPAC-QOL	Japanese Version of the Patient Assessment of Constipation Quality of Life
LCMS	Liquid Chromatography/Mass Spectrometry
LPS	Lipopolysaccharide Binding Protein
LA	Locomotory Activity
FMI	Multi-Dimensional Fatigue Inventory
MNQI	Methyl-Donor Nutritional Quality Index
MSQ-BR	Mini Sleep Questionnaire
MWM	Morris–Water Maze
MDA	Malondialdehyde
MGBA	Microbiota–Gut–Brain Axis
N3	Stage 3 Non-Rapid Eye Movement Sleep (Slow-wave sleep)
NREM	Non-Rapid Eyes Movement Sleep
NORT	New Object Recognition Test
OFT	Open Field Test
OSQ	Oviedo Sleep Questionnaire
OSA-MA	Ogri–Shirakawa–Azumi Sleep Inventory MA version
OLT	Object Location Memory Test
PSQI	Pittsburgh Sleep Quality Index
POMS2	Profile of Mood State Second
PCPA	p-Chlorophenylalanine
*Per1/Per2*	Period Circadian Protein 1/Period Circadian Protein 2
PRISMA	Preferred Reporting Items for Systematic Reviews and Meta-Analyses
PSG	Polysomnography
PSS	Perceived Stress Scale
REM	Rapid Eyes Movement Sleep
RCT	Randomized Controlled Trial
RoB 2.0	Cochrane Risk of Bias Tool (Version 2)
rRNA	Ribosomal Ribonucleic Acid
RT-qPCR	Reverse Transcription Quantitative Polymerase Chain Reaction
SDS	Self-Rating Depression Scale
SAS	Self-Rating Anxiety Scale
STAI	State Trait Anxiety Inventory
SRBSQ	Sleep Related Safety Behavior Questionnaire
SSR	Sleep Self Report
SM	Sympathetic–Adreno–Medullary Axis
SAM	S-adenosylmethionine
SCFA	Short Chain Fatty Acid
SS	Symptoms Severity Scale
SPT	Sucrose Preference Test
SCFA	Short-Chain Fatty Acid
STYHCD	Traditional Chinese Medicine Decoction
SYRCLE	Systematic Review Centre for Laboratory Animal Experimentation (Risk of Bias tool)
TCM	Traditional Chinese Medicine
TST	Tail Suspension Test
T-SOD	Total Superoxide Dismutase
TNF-α	Tumor Necrosis Factor-alpha
TSS	Tiredness Symptoms Scale
TM	T-maze
VAS	Visual Analog Scale
VAMS	Visual Analog Mood Scales
WASO	Wake After Sleep Onset
WPI	Widespread Pain Index
YM	Y-maze
