# Supplementary material for: Gut Microbiota Modulation as a Therapeutic Strategy for Insomnia: A Systematic Review of Nutritional and Botanical Interventions
Source: Biomolecules. 2026 Jun 23;16(7):933. doi: 10.3390/biom16070933 (PMC13406307; doi:10.3390/biom16070933)
Supplement: Supplementary file 1 [file biomolecules-16-00933-s001.zip › Table S1-S4.pdf]

Supplementary Table S1 Summary of clinical studies involving the roles of nutritional and herbal interventions on gut microbiota modulation as a therapeutic strategy for the treatment of insomnia.

Ref.	Study design	Country/ Ethnic	intervention	Objectives	Methods	Main findings	Conclusion
[63] Swada et al., 2019	Randomized double-blinded, placebo-controlled; 12-wk intervention in healthy long-distance running athletes (N=49)	Japan	Probiotics Lactobacillus gasseri heat-inactivated CP2305 vs. Placebo beverage	To investigate the beneficial effects of para-probiotics in athletes experiencing physical & mental stress.	Mental state questionnaire (CFS, STAI, HADS, PSQI). Stress biomarkers (Salivary cortisol, Chromogranin A). Fecal microbiota (16S-rRNA sequencing)	CP2305: decrease in scores of CFS, STAI-trait & HADS; significant reduction in chromogranin A; no change in salivary cortisol & effectiveness in sleep quality; no significant change in alpha & beta-diversity; significant increase in Faecalibacterium.	CP2305 (12 wk): amelioration of stress-associated physical & psychological long-distance runner athletes based on self-report mental state questionnaire & chromogranin A (despite no change in richness & diversity of gut microbiota); significant gain of Faecalibacterium composition, preventing reduction of *Bifidobacterium* (both: significant role in gut health & homeostasis).
[45] Butler et al., 2020	Single-arm 12-wk intervention in healthy participants (N=24)	Ireland	Dietary change farm-based cookery course vs. unpasteurised milk (12 wk)	To investigate the impact of dietary change (unpasteurized milk) on gut microbiome, psychological status & metabolites.	Sleep quality measurement (PSQI). Stress markers (PSS, HADS, IPAQ). Fecal microbiota (16s rRNA, SCFAs).	No change in psychological status, alpha-diversity & beta-diversity; positive correlation between *Lactobacillus* abundance vs. unpasteurized milk vs. dairy product; significant increase in SCFA.	Unpasteurized milk: increase in abundance of Lactobacilli after 12 wk in healthy participants, but no change in psychological states, possibly due to healthy status.
[22] Valle et al., 2021	Randomized, placebo-controlled; 30-day intervention in military training students (N=65)	Brazil	Synbiotics in ice cream (Inulin, L. Acidophilus LA-5 & B. Animals BB-12) vs. placebo	To evaluate the safety & efficacy of symbiotic ice cream on salivary IgA, gastrointestinal symptoms, well-being & gut microbiota.	Sleep quality measurement (PSQI). Mental status (VAS mood & well-being questionnaire). Fecal microbiota (16s rRNA, ammonia, SCFAs). Safety (salivary IgA).	Symbiotics: increase in tenseness & sleepiness; decrease in PSQI (<5); no significant difference in alpha-diversity but higher Firmicutes; greater relative abundance of some genera.	Synbiotics ice-cream: improvement of sleep quality and lower tenseness in healthy military training students facing extreme conditions (physical & mental challenge); potential to change in gut composition.
[64] Schaafsma et al., 2021	Randomized controlled, cross-over; 3-wk intervention	Netherland	Prebiotics vs. Dairy-based product + Galacto-oligosaccharides (GOS)	To evaluate the efficacy & safety of a dairy-based product (DP) containing prebiotics on sleep quality & stress.	Sleep quality assessment (PSQI, REM, NREM, WASO). Psychological assessment (DASS-42). Fecal microbiota (16s rRNA).	DP group: significant reduction of PSQI score (day 14); 10 minutes longer REM sleep; lower cortisol (day 21); increase in relative abundance of Bifidobacterium.	DP: no significant effect on sleep in healthy adults with sleep problems after 3 wk; spontaneous improvement in sleep quality in some; lowering

	in healthy subjects with sleep problems<br>(N=70)				Stress biomarkers (saliva cortisol).		early-morning cortisol & increasing Bifidobacterium despite no change in psychological stress.
[39]<br>Colombo et al., 2021	Randomized, double-blinded; 16-wk intervention<br>in infants (N=116)		Prebiotics vs.<br>Cow's milk-based infant formula with prebiotics blend polydextrose vs. palactooligosacharide (PDX/GOS) vs. Cow milk-based infant formula	To evaluate the effect of a prebiotics blend added to cow's milk-based infant formula on sleep-wake patterns in infants.	Sleep-wake report (infant behavior diary).<br>Sleep measurement (Actigraphy: wearable actigraph device).<br>Stress biomarker (saliva cortisol).<br>Fecal microbiota (16s rRNA).	PDX/GOS group: significant decrease in waking (crying & fussing) on day 112; longer diurnal nap duration (day 70); no difference in sleep duration; no significant change in alpha & beta diversity;<br>increase in Lachnospiraceae in the prebiotic group (day 112).	Prebiotics: shortening the episode of crying & daytime nap duration at a specific time point; improvement of behavioral indicator of tolerance in infants.
[52]<br>Kobayashi et al, 2022	Single-arm, open-label pilot study in females with GI symptoms & anxiety<br>(N=21)	Japan	Kamikihito kampo medicine, containing 12 herbal medicines	To evaluate the safety & efficacy of Kamikihito on psychological & gastrointestinal symptoms.	Mental status (POMS2, STAI, GSRS).<br>Stress biomarkers (Oxytocin, Orexin, Vasopressin).<br>Fecal microbiota (16s rRNA, metabolites).	Kamikihito: significant decrease in POMS2; improvement of STAI score, TMD & DD sub-scale of POMS2; significant decrease in bioactive substance with a strong association with anxiety score; no significant change in gut microbiome composition.	Kamikihito: safe & effective for treatment of psychological & gastrointestinal symptoms in female patients.<br>Association between marked increase in plasma metabolites vs. depression.
[43]<br>Baldi et al., 2022	Randomized, double-blinded, cross-over; 24-wk intervention<br>in Fibromyalgia (FM) patients (N=20)	Italy	Khorasan wheat diet vs. control wheat diet	To evaluate the effect of Khoisan wheat diet on mental status, sleep, gut microbiome & metabolites.	Sleep quality (SRSBQ, FOSQ).<br>Well-being questionnaires (WPI, SS, FIQ, TSS).<br>Fecal microbiota (16s rRNA, SCFAs).	Khoisan wheat: no significant difference in alpha & beta diversity; significant increase in Saccharibacteria & Actinobacteria; decrease in Enterococaceae;<br>no significant change in SCFA.	Khoisan wheat: modification of gut composition & function with improvement in Fibromyalgia patients.
[41]<br>Lawrence et al., 2022	Pilot, non-randomized feasibility study<br>in non-medicated Attention Deficit Hyperactivity Disorder (ADHD) children (N=9)	UK	Dietary intervention targeting gut microbiota	To assess the feasibility of dietary intervention to support future randomized clinical trials.	Sleep quality measurement (Actigraphy's motionlogger micro-watch, sleep diary, CSHQ-A, SSR).	Modest change in alpha diversity score from T1 & T2 in family of Lachnispiraceae, Roseburia & Blautia, Bifidobateriaceae, Sutterella, Ruminococcaceae & Bacteroides.<br>Actigraphy: tendency for improvement in sleep quality & minimal change in questionnaire.	Feasibility for further RCT trial in ADHA children.<br>Dietary intervention targeted the gut microbiome: tendency toward improvement in sleep actigraphy & sleep score questionnaire.

[36] Makela et al., 2023	Randomized, triple-blinded, placebo-controlled; 10-wk intervention in healthy students facing examination stress (N=190)	Ireland, UK	Probiotics Lacticaseibacillus paracas Lpc-37	To evaluate the efficacy & safety of Lacticaseibacillus paracas Lpc-37 on stress reduction and change in state of anxiety.	Mental state questionnaire (STAI from Y-1 & 2, DASS-21, HADS, PSS, BL-VAS, VAS, PSQI).<br>Stress biomarkers (salivary cortisol)<br>Fecal microbiota (16S-rRNA)	Lpc-37: no significant change in stress, depression, anxiety & sleep quality (questionnaire & salivary cortisol), alpha & beta diversity.<br>Severe AEs (abdominal discomfort & nausea) in 14 participants (12 from the study group, 2 from placebo).	Lpc-37: no significant change in stress, anxiety, depression & sleep quality; no change in gut microbiota composition & diversity in a group of students under examination stress (possibly due to inadequate change in mental state perception of stress in university students).
[37] Mysonhimer et al., 2023	Randomized controlled cross-over; 4-wk intervention in healthy subjects (N=28)	USA	Prebiotics Fructooligosaccharides (FOS) & Galactooligosaccharides (GOS)	To investigate the effects of prebiotics FOS & GOS on metal biological markers of stress, inflammation & mental health symptoms.	Stress biomarker (urinary free cortisol: UFC).<br>Inflammation biomarkers (TNF-alpha, LPS, CRP, AGP, IL-6).<br>Mental health assessment (DASS-42, Emotionally arousing images).<br>Sleep quality assessment (ActiGraph wrist-worn accelerometer device).<br>Fecal microbiota (16s rRNA).	FOS & GOS: no change in biological markers, sleep quality & mental health, or emotional symptoms;<br>increase in pecan sequences of phylum Actinobateriota & genus Bifidobacterium; greater abundance of Bifidobacterium; lower Lactococcus lactic, Dielma sap. & Adsiella dolichum; no change in alpha or beta diversity.	FOS & GOS: modulation of gut microbiota, especially Bifidobacterium, but no change in mental-related biomarker, stress, emotion & sleep quality.
[51]] Crichton et al. 2023	Randomized, double-blinded, placebo-controlled; 2-wk intervention in healthy adults (N=51)	Australia	Ginger supplement, nonsynthetic ginger (Zingiber officinale)	To evaluate the safety & efficacy of ginger root powder on gut flora composition, GI symptoms & mental status.	Mental status questionnaire (DASS-21, MFI, VAS EQ-5D-5L).<br>Gut health (GSRS).<br>Fecal microbiota (16s rRNA).	Ginger supplement: change in relative abundance in placebo compared to post ginger intervention; greater abundance of Parabacteroides, Bacillus, Ruminococcaceae incite seeds, unclassified Bacilli, Deflu vitaleaceae, Morganellaceae & Bacillaceae.<br>No AE.	Ginger supplement: potential for alteration in gut microbiota composition even without significant change in alpha & beta diversity & mental/mood status.
[65] Li et al. 2024	Randomized, triple-blinded, placebo-controlled; 28-d intervention in healthy adults with high mental stress, insomnia, overweight & constipation	China	Probiotics *Bifidobacterium breve* 207-1 vs. placebo	To evaluate the safety & efficacy of *Bifidobacterium breve* 207-1 on specific neurotransmitters, hormones & ability to regulate lifestyle behaviour.	Smart watch data (daily sleep).<br>Questionnaires (SDS, SAS, PSQI).<br>Stress biomarkers (5-HT, GABA, CRH, ACTH, cortisol).<br>Fecal microbiota (16S-Rrna, SCFAs).	High-dose: significant decrease in ACTH, PSQI score, alpha & beta-diversity; low F/B ratio.<br>Low dose: significantly lower CRH.<br>Depression & anxiety: no significant difference in each group before and after intervention.<br>Placebo: significantly higher relative abundance of *Desulfobacterota.*	B.Breve 207-1: increase in GABA; stabilization of 5-HT; decrease in cortisol; potential for alteration of gut microbiomes.<br>Change in neurotransmitter & hormone levels: not necessarily lead to mood change, requiring prolonged use.

	(N=120)						
[17]<br>Li et al. 2024	Randomized, triple-blinded, placebo-controlled; 28-d intervention in healthy adults with high mental stress, insomnia, overweight & constipation(N=120)	China	Probiotics<br>*Lacticaseibacillus paracasei* 207-27	To evaluate the safety of *Lacticaseibacillus paracasei* 207-27 on sleep.	Smart watch data (daily sleep EEG, PSG).<br>Questionnaire (PSQI).<br>Stress biomarkers (5-HT, GABA, CRH, ACTH, cortisol)<br>Fecal microbiota (16S-rRNA, SCFAs).	Increase in sleep duration & PSQI score; significant decrease in ACTH, COR, CRH with low-dose; no change in neurotransmitters, alpha diversity & gut composition; significant increase in Lactobacillus & all SCFAs in low-dose group except isobutyrate & isovalerate; no AE.	L. Paracasei 207-27: potential to improve sleep quality & regulation of gut microbiota community & metabolites in healthy adults.
[40]<br>Lozar Kriver et al. 2024	Randomized, double-blinded, placebo-controlled;<br>6-wk intervention in neonates treated with antibiotics<br>(N=89)	Slovenian	Probiotics<br>*Limosilactobacillus reuteri* DSM17938	To investigate the effects of probiotics on the development of functional gastrointestinal disorder, crying & sleep duration in neonates exposed to antibiotics in early life.	Behavioral evaluation (daily sleep & crying duration, IGSQ).<br>Fecal microbiota (16S rRNA).	Probiotics: shorter sleep & crying duration: shorter in the probiotics group; no change in Lactobacilli, *Bifidobacterium* & Enterobacteriaceae groups; higher strain-specific detection of L. reuteri DSM 17938 (6 wk); significantly greater abundance of Lactobacillus (6 months).	Single probiotic intervention in term neonatal subjects during & after: increase in sleep duration; limited effect on FGID symptoms; no change in gut microbiota composition but increase in abundance of Lactobacillus; potential to minimize the negative impact of antibiotics on gut function; worth further investigation on early life exposed to antibiotics.
[15]<br>Tian et al. 2024	Randomized, double-blinded, placebo-controlled; 4-wk intervention in insomnia patients<br>(N=60)	China	Probiotics<br>*L. Helveticus* CCFM1320	To investigate the effects of probiotics on mitigating insomnia symptoms.	Sleep quality measurement (AIS, PSQI).<br>Sleep biomarkers (SAMe).<br>Fecal microbiota (16s rRNA).	Probiotics: significant decrease in PSQI score, especially in high-dose; no difference in AIS; significant increase in serum SAMe (high dose);<br>significant increase in SAM & cortisol (high-dose); decrease in cortisol; no significant difference in alpha & beta-diversity; significant increase in abundance of Lachnospiraceae ND3007.	Potential improvement of the PSQI score of sleep with high dose L. Helveicus CCFM1320 without altered composition of gut microbiota diversity; promotion of probiotics and reduction of pathogenic species.

[35] Kortman et al. 2024	Randomized, cross-over; 3-wk intervention in healthy subjects with moderate sleep disturbance (N=67)	Netherland	Prebiotics dairy product (DP) with GOS, tryptophan, vitamins, and minerals vs. placebo	To investigate the effects of GOS on gut microbiota, the pathways underlying its interaction with the brain based on gut-brain modules (GMBs).	Sleep quality assessment (PSQI). Fecal microbiota (shotgun metagenomics), organic acid (HPLC).	DP-GOS: no significant difference in alpha & beta diversity & PSQI score; increase in Bifidobacterium. Higher Faecalibacterium prausnitzi I and lower in Bifidobacterium at baseline in responders; a significant increase in Bifidobacterium only in the responder group after intervention.	DP-GOS: small change in gut microbiota & baseline gut microbiota, which determine favorable benefit for sleep quality improvement.
[50] Gillies et al. 2024	Randomized, double-blinded, placebo-controlled, cross-over; 4-wk intervention in healthy females (N = 38)	New Zealand	Flavonoid-rich blackcurrant beverage	To investigate the effects of flavonoid-rich blackcurrant beverage (FBB) on mental health, gut microbiome composition, cognitive function & mood.	Metal status questionnaire: STAI; Bond-Lafer mood scale; VAMS; Stress & fatigue VAMS, POMS. Sleep quality (PSQI). Gut-brain axis biomarkers: Kynurenine, Tryptophan, BDNF, IL-6. Stress & cognitive: Purple MTF. Fecal microbiota (shotgun metagenomic).	FBB: no significant change of stress reactivity & scores; significant improvement of cognitive performance in letter retrieval; difference in mood change on the POMS questionnaire; no change on the PSQI. No difference in alpha diversity; increase in abundance of Bifidobacterium spp. in responder group (improvement of cognitive function).	Daily FBB consumption: improvement of cognitive function, mood & alteration of gut microbiota genera but not composition.
[23]Meng et al, 2025	Cross-sectional, Chinese (18-65 yr, N=650)	China	Dietary Index for gut microbiota (DI-GM)	To investigate the association between DI-GM with depression, anxiety, and intestinal tumor biomarkers, and to explore whether gut microbiota mediates the association.	Dietary assessment: DI-GM score. Psychological assessment: PHQ-9; STAI; PSQI; WHO-5. Fecal sample: 16s rRNA gene sequencing. Biomarkers: CRP; BDNF; TMAO; CEA; fecal calprotectin.	PHQ-9 & STAI: significant improvement in depression & anxiety in high DI-GM score. PSQI: correlation between improved vs. improved DI-GM scores. High DI-GM: impact on specific taxa than overall diversity loss (decrease in Shanon Index). DI-GM: consistent linkage with reduces systemic & intestinal inflammation.	Significant association between the Dietary index for Gut microbiota (DI-GM) and inflammatory biomarkers & mental health.
[18]**Tanihiro et al, 2024**	Randomized, double blind, Placebo-controlled in healthy Japanese adults (22-64 yr, N=40).	Japan	Yeast manna (YM)	To evaluate the efficacy & safety of yeast manna in improving bowel habit & sleep quality, along with metabolic in fecal samples.	Sleep assessment: EEG (TIB); SE); N3 duration; SOL; N3 latency; WAKE; REM; NREM. Bowel habits assessment: BSS. Fecal sample: 16s rRNA gene sequencing, metabolites. Adverse events.	YM: significant prolongation of TIB; shortening of N3 latency; improvement of defecation habits with increase in stool volume. No significant change in gut microbiota numbers. Differential fecal metabolites between two treatment.	YM: significant prolongation of total time bed and shortening of sleep latency, mediated by propriotnate & GABA levels.

[24] Zeng et al., 2024	Interventional clinical trial in insomnia patient with TCM syndrome (N=63)	China	Center-supplementing + qi-boosting decoction (CSQBD) vs. spleen-tonifying + yin heat-clearing decoction (STYHCD)	To evaluate the effect of CSQBD & STYHCD in treating insomnia patient with spleen qi deficiency & stomach heat (TCM syndrome)	Sleep assessment: PSQI; ISI.<br>Fecal sample: 16s rRNA gene sequencing.<br>Stress&nflammatory biomarkers: IL-6; IL-1β; TNF-α; TNF-β; IFN-α; IL-10;cortisol	Both herbal formula: significant improvement of PSQI & ISI scores.<br>Significant difference in beta diversity or gut microbiome profiles over the intervention period. Bactericides coprophilus: significant enrichment in both treatments.<br>CSQBD: reduction of plasma cortisol levels & increase in anti-inflammatory cytokine IL-10 with reduction of IFN-α.	The study suggested TCM syndromes and insomnia symptom alleviate by Chinese herbal formula and can be mediated by some gut microflora that impact on host immune response.
[47]<br><br>Jiang et al., 2024	Cross-sectional, observational (N=178)	Chinese	Dietary habits associated with gut microbiota among individuals with sub-threshold depression (SD)compared with healthy subjects	To investigate the association between dietary habits and gut microbiota composition among individuals with subthreshold depression.	Mental status questionnaires (SDS, SAS, BDI-II).<br>Depression scale: HAMD-17.<br>Sleep quality: PSQI.<br>Fecal microbiota: 16S rRNA, shotgun metagenomics.	DP-I consumption: reduction of risk of sub- threshold depression (SD).<br>DP-IV consumption: increase of the risk of having SD.<br>SD group: higher community richness compared with healthy; 15 taxa enriched, including Faecalibacterium prauanitzii, Eubacterium, and Agathobaculum butyriciproducens; difference in genus level between the two groups.<br>Correlation between diet vs. bacterial taxa.<br>SDS, SAS, BD-II, PSQI scores: positive correlation with specific microbial species.	SD: more richness in gut microbiota with correlation align with dietary pattern; correlation between specific gut microbiome species and mental status score.<br>Linkage between dietary patterns and SD suggests that dietary patterns and gut flora might hold the key as therapeutic targets for SD prevention or treatment in the future.
[20]<br><br>Lau et al. 2024	Non-randomized open-label, prospective; 12-wk intervention in Post-acute COVID-19 syndrome (PACS)<br>(N=60)	China	Fecal microbiota transplantation(FMT), fecal donated from 2 healthy unrelated donors chosen based on gut profile with high relative abundance of G formicilis	To evaluate the safety & efficacy of FMT on alleviating PACS insomnia symptoms.	Sleep quality assessment (ISI, PSQI, ESS).<br>Psychological assessment (GAD-7).<br>Fecal microbiota (shotgun metagenomics).<br>Stress biomarkers (cortisol, melatonin).	FMT: significant decrease in PSQI score & daytime sleepiness ESS score; improvement of GAD-7 score; significant decrease in cortisol; improvement in gut microbiota richness at the species level G formicillis & Coprococcus comes.	FMT: effective & safe treatment for post-COVID-19 insomnia & anxiety with potential improvement of sleep quality and modulation of gut microbiota.

[46]<br>Chen et al. 2024	Cross-sectional observation in elderly adults from the TALENTs trial<br>(N=301)	China	Methyl donor nutrient (Vit B6, B12, folate, choline)	To investigate the association between methyl donor nutrients (MDN) dietary intake & sleep disorder (SD) & gut composition in the elderly.	Pre-trial PSQI assessment (PSQI>=5).<br>Dietary intake (PAD, MNQI).<br>Fecal microbiota (16s rRNA).	Sleep-deprived group: significantly lower MNQI.<br>SD: Lower consumption of MDNs.<br>Alpha-diversity: no significant difference.<br>Beta diversity: lower in the SD group compared with the control.<br>Relative abundance of 5 genera: positive correlation with PSQI score; negative correlation with MNDs intake.<br>Anoxybacillua: opposite pattern.	Potential association between MDN consumption and sleep quality & diversity of gut microbiota in the elderly.
[21]<br>Guan et al. 2025	Randomized, double-blinded, placebo-controlled; 2-wk intervention in pre-graduated students<br>(N=120)	China	Probiotics<br>*Lacticaseibacillus paracasei* K56 vs. Placebo	To evaluate the safety & efficacy of *Lacticaseibacillus paracasei* K56 on perceived stress.	Mental measurement (PSS-10, DASS-21, ISI, fatigue severity scale).<br>Stress biomarkers (5-HT, cortisol, IL-1β, IFN-gamma).<br>Fecal microbiota (shotgun metagenomics, non-targeted LC-MS based metabolomics).	Probiotics: greater decrease in PSS-10 score, DASS-21, ISI; pronounced effect on sleep quality among females; no significant change in alpha & beta diversity & K56; significant increase in Lacticaseilbacillus parase; increase in 5-HT.<br>Negative correlation in the increase of the relative abundance of Lactobacillus and a higher score of the mental state questionnaire.	Probiotics: potential benefit of Lacticaseibacillus paracsei K56 in amelioration of stress, anxiety & insomnia together with an increase in beneficial gut microbiota & butyrate in master's & doctoral students' graduation-related stress.
[16]<br>Wang et al. 2025	Randomized, placebo-controlled;<br>8-wk intervention in healthy subjects<br>(N=50)	China	Probiotics<br>*Lactobacillus delbrueckii* subs. *Bulgaricus* LB42 vs. Placebo	To evaluate the safety & efficacy of *Lactobacillus delbrueckii* subs. *Bulgaricus* LB42 on gastrointestinal function, sleep quality & faecal calprotectin	Sleep measurement (PSQI).<br>Gastrointestinal health (GSRS, BSS).<br>Safety biomarkers (IL-37, calprotectin, IgA, IgE).	Probiotics: significant decrease in PSQI; improvement of GI health; no significant change in alpha-diversity but increase in abundance of Bacteroidota & enrichment of predominantly in members associated with fermentative metabolism & SCFA production.	Lactobacillus delbrueckii subs. Bulgaricus LB42: safe; significant improvement of sleep quality in healthy; improvement of gut health despite no change in overall gut microbiome composition; potential to enrich SCFA-producing genera.
[38]<br>Santamarina et al. 2025	Randomized, double-blinded; 90-d intervention in healthy participants<br>(N=91)	Brazil	Natraceutical with prebiotics vs. Silymarin seed extract (NSupple silybum) vs. control prebiotic without Silymarin seed extract (NSupple)	To investigate the effects of a nutraceutical blend on gut microbiota shaping, body composition & sleep quality.	Sleep quality assessment (MSQ-BR, ESS, PSQI, WHOQoL-BREF).<br>Mental state assessments (BRUMs).<br>Fecal microbiota (16s rRNA), plasma Sylmarin (HPLC).	NSupple: significant improvement in ESS score; decrease in PSQI score; no significant change in MSQ-BR & BRUMs; decrease in Bacteroidetes & increase in Firmicutes; decrease in F/B ratio.<br>NSuppleSilybum: improvement of WHOQoL-BREF in general health; increase in Bacteroidetes; decrease in F/B ratio; reduction in *Alistipes indistinctus, Blautia*	Nutraceutical blend: modest alteration of gut composition & homeostasis; improvement of sleep quality in the group without Silymarin seed extract.

						obeum, Haemophilus parainfluenzae, Ruminococcus lactaris & *Victi-Vallis Vadensis*.<br>No significant change in beta-diversity between groups & time points.<br>Reduction of NSupple_Silybum alpha-diversity indices: Pielou's evenness & Simpson index.	
[27] Arce-Lopez et al. 2025	Randomized, double-blinded, placebo-controlled;<br>6-wk intervention<br>in healthy adults (N= 58)	Mostly of Mediterranean European descent	Fiber-enriched kombucha vs. Unfermented tea	To investigate the effects of fiber-enriched kombucha on biochemical parameters & gut microbiota composition.	Mental status questionnaire (DASS-2).<br>Sleep quality measurement (OSQ).<br>Gut health (GSRS).<br>Fecal microbiota (16s rRNA).	Fiber-enriched kombucha: no significant change in DASS-2 & OSQ; significant change in gut microbiota composition (*Bifidobacterium* most increased, *Rummiococcus torques* most decreased).	Short-term consumption of fiber-enriched kombucha: effect on lipid level & alteration of gut composition in healthy adults.
[44] Inoue et al. 2025	Randomized, double-blinded, controlled; 4-wk intervention in healthy adults (N=58)	Japan	High-fibre diet (HiFib) vs. Low-fibre diet (LoFib)	To investigate the effects of high- vs. low-fibre diet on gut microbiota, bowel-related quality of life, sleep & skin condition.	QOL Questionnaire (JPAC-QOL)<br>Sleep quality measurement (OSA-MA).<br>Fecal microbiota (16S rRNA).	High-fiber: significantly higher in *Bifidobacterium* 388775 (wk 2 & 4); significantly lower JPAC-QOL; no difference in alpha diversity; significantly higher weighted UniFrac distance.<br>*Anaerostipes* & *Fusicatenibacter*: positive correlation with OSA-MA after correlation test.	High-fiber diet: potential to modulate gut microbiota composition, improvement of bowel-related quality of life within 4 wk, but limited change in sleep (possibly due to short time frame).
[48] Lane et al. 2025	Randomized controlled; 3-wk intervention in healthy high body mass index women<br>(N=47)	Australia	Food-based diet with very low energy (VLED) vs. Supplement-based VLED	To evaluate the effect of food- versus supplement-based VLED program on gut microbiome composition, serum biomarker, mental health, sleep & gastrointestinal symptoms.	Psychological assessment & QOL (DASS-21, WHO-5).<br>Sleep quality measurement (AIS).<br>Fecal microbiota (Shotgun metagenomics).	Food-based VLED: higher alpha diversity & richness; significant differential change of beta diversity; no change in mental status or sleep quality.	Both interventions: weight-loss promotion.<br>Food-based VLED: significantly greater in diversity & richness; some shift in beta diversity (more favourable improvement of gut microbiome composition compared to supplement-based).<br>AE: headache.
[	Randomized, open-labelled, cross-over; 8-	USA	Immediate vs. delayed intervention (higher	To evaluate the effect of lifestyle modification by increasing	Sleep assessment (Garmin device for sleep & activities assessment).	Carbohydrate: no significant effect on body composition; higher RANS-SF-12 score in immediate	Challenges of lifestyle interventions for night-shift workers to modify dietary intake & sleep patterns:

[13]Robinson et al. 2025	wk lifestyle intervention in female healthcare night shift workers (N=13)		carbohydrate, lower fat meal at different times in the morning & evening + whey protein 2 times per day)	carbohydrate intake in the earlier active period on mitigating the metabolic & psychological effects.	QOL (RANS-SF-12).<br>Body composition (Height, BMI, Lean body mass, Visceral fat mass)<br>Inflammatory biomarkers (IL-1β, TNFα).<br>Endotoxin biomarker: (LBP)<br>Fecal microbiota (16s rRNA).	intervention group; no change in mental component scores; longer nightly sleep & rest hours in the immediate group; no difference in alpha & beta diversity.	improvement of metabolic health, emphasizing the need for a multifaceted approach.
[28] Sasaki et al.2025	Randomized, single-arm, single -blind; 8- wk intervention in healthy subjects (N=99)	Japan	Prebiotics vs. Personalized granola (Inulin, barley, FOS, GOS, resistant starch, Hi-cacao)	To evaluate the effects of personalized granola prebiotics on gut microbiota, SCFA production, metabolic & mental health.	Mood state assessment (POMS2).<br>Sleep quality assessment (AIS).<br>Fecal microbiota (16S rRNA).	SCFA: trend toward an increase.<br>Relative abundance of Bifidobacterium: significant increase across participants.<br>POMS: significant increase (wk 4) & decrease in total mood-disturbance score (wk 8).	Personalized granola formulated according to individual gut microbiota genera: modest boost of SCFA production, increase in Bifidobacterium & improvement of overall mood disturbance.

[42]]<br><br>{Meng, 2025 #430}	Cross-sectional, observational in Colon cancer patients (N=603)	China	Mediterranean-DASH Intervention for Neurodegenerative Delay (MIND diet) & Dietary Index for gut microbiota (DI-GM)	To investigate the association of MIND and DI-GM scores with depression, anxiety, sleep quality, quality of life & inflammatory markers.	Psychological assessment (HADS).<br>Sleep quality assessment (PSQI).<br>QOL (FACT-C)<br>Fecal microbiota (16S rRNA)<br>Inflammatory Biomarkers (CRP, IL-6, Calprotectin).<br>Neuroplasticity brain health biomarker(BDNF)	Higher MIND score associated with lower HADS-A & HADS-D and higher FACT-C.<br>Increase in MIND scores: relationship with increased alpha-diversity & decreased F/B.<br>Higher DI-GM score: lower BMI, improved HADS, QOL, PSQI & alpha diversity.<br>Higher MIND & DI-GM: higher serum BDNF.<br>F/B ratio: association between DI-GM & depression.<br>Gut composition: partly beneficial effect on psychological symptoms.	Greater adherence to MIND & DI-GM dietary pattern: better QOL, psychological outcomes, sleep quality, higher BDNF, emphasizing the role of gut microbiota as a mediator linking diet to mental well-being.

[49]<br><br>{Tao, 2025 #208}	Cross-sectional (N=100)	China	Vitamin B1 & B2	To examine the relationship among mental health indicators, vitamin B1 & B2 levels, and gut microbiota.	Psychological assessment (SAS, PSS).<br>Sleep quality assessment (PSQI, ESS).Urinary vitamin B1, B2.<br>Fecal microbiota (16s rRNA).	No significant difference in SAS or PSS across groups with varying vitamin B1.<br>PSQI & ESS scores: significant difference between groups.<br>Vitamin B2: association with sleepiness ESS score.<br>Vitamin B1: positive correlation with PSS score; negative correlation with PSQI.<br>Vitamin 2: negative correlation with ESS score; no correlation with others.<br>C. Butyricum, Bacteroides & Enterococcus: negative correlation with higher sleep quality.<br>Enterobacteriaceae: significant difference between free vs. moderate anxiety group.<br>Free anxiety group: more diverse gut microbiota than anxiety group.	Association between vitamin B1 and B2 levels, specific gut microbes, and individuals' anxiety, stress, sleep quality & sleepiness.<br>Higher vitamin B1: better sleepiness & higher vitamin B2 linked to better sleepiness.<br>Significant correlation between specific gut microbes vs. host anxiety, sleep quality & sleepiness.

Supplementary Table S2 Summary of *in vivo* studies involving the roles of nutritional and herbal interventions on gut microbiota modulation as a therapeutic strategy for the treatment of insomnia.

Ref	Study design	Type of intervention	Objective	Methods	Main findings	Conclusion
[53] Thompson et al. 2016	Experimental study in male F344 rats (N=52)	Diet containing prebiotics GOS & PDX, Lactoferrin (Lf) & milk fat globule membrane (MFGM) vs. Calorie-matched control diet	To investigate the protective effect of prebiotics on stressor exposure-related gut alteration & sleep/wake cycle.	Sleep assessment (F40-EET Biotelemetry transmitter device implantation, EEG, NREM, REM, WAKE time, CBT, LA). Fecal microbiota: 16s rRNA, Selective culture of *Lactobacillus rhamnosus* to measure colony-forming unit (CFU).	Test diet: effective increase in *Lactobacilli rhamnosus;* improvement of NREM sleep consolidation when treated early in life (involved in anxiety & depressive-like behaviour reduction); enhancement of REM rebound during the dark after stress exposure; diurnal amplitude of CBT; no significant shift in high-abundance phyla across development; significant interaction between diet vs. stress in the number of observed species diversity.	Prebiotics: improvement of early life NREM sleep; enhancement of REM rebound or promotion of sleep recovery after exposure to stress; protection from stress-induced decrease in gut microbial alpha diversity without change in microbial community.
[54] Thompson et al. 2020	Experimental study in F334 rats (N=52)	Diet containing prebiotics GOS & PDX, Lactoferrin (Lf) & milk fat globule membrane (MFGM) vs. Calorie-matched control diet	To investigate if stress and/or dietary prebiotics (Test diet) alter the fecal metabolome, and explore their association with sleep and/or gut microbial alpha diversity.	Sleep assessment (F40-EET Biotelemetry transmitter device implantation, EEG, NREM, REM, WAKE time). Fecal microbiota (metabolome: LC/MS).	Test diet: significantly higher 20 metabolites.<br>Stressor: increase in allopregnanolone precursor & ketone steroid (family of endogenous metabolites of corticosteroid/progesterone).<br>Diet & stress: significant difference between groups in 36 metabolites after exposure to stress; higher levels of 28 metabolites in the test diet group with no effect of stress; change in levels of 8 metabolites by both diet and/or stress.<br>Additional family members: 5. alpha.-Pregnane-3.alpha., 21-diol-20-one, better known as allotetra-hydrodeoxy corticosterone (potentiates GABAergic inhibition & linked to sleep quality reduction and involved in acute stress response).<br>Pyrimidine nucleotide: significant linear relationship with NREM sleep.<br>Ketone steroid & Ethanebis(thioate) derivative: association with REM sleep after stress exposure.	Dietary prebiotics: alteration of gut microbial composition & production of metabolites, alleviating stress in sleep disruption; assistance in preserving the diversity of normal flora.<br>Stress: modulation of neuroactive steroid signalling in the gut, attenuated by a prebiotic diet.<br>Microbial-dependent metabolites: role in the gut-brain axis through improvement of NREM/REM.

[66] Yu et al. 2020	Experimental study in 6-wk ICR mice (N= 60)	Milk fermented with Lactobacillus braves DL1-11 (High GABA producing chain)	To evaluate the effect of GABA fermented milk on anxiety & sleep quality.	Stress assessment (OFT).<br>Anxiety assessment (EPM).<br>Sleep assessment (Sleep improvement test).<br>Hypnotic effect assessment (PBIT).<br>Fecal microbiota (16S rRNA, SCFAs).	High GABA fermented milk (HGFM): significantly higher time & frequency spent in the central area; significantly higher time entered in open arms; effect of GABA-rich fermented milk on sleep (shortening of sleep latency & prolonged pentobarbital-induced sleep duration); significant change in beta-diversity; increase in relative abundance of Ruminococcus, Adlerbaculum, SCFA.	L. Braves DL1-11 at different doses: different outcomes of psychological & gut microbiomes.<br>High-dose GABA-fermented milk: effective reduction of stress & anxiety; promotion of sleep; modulation of microbiota gut composition & metabolites in mice.
[26] Thompson et al., 2021	Experimental study in SD rats (N= 84)	Diet containing prebiotics GOS & PDX (ad libitum) vs. Calorie-match control diet	To evaluate the effect of prebiotics on physiology & sleep/wake behaviour in an animal model under stress-induced light/dark cycle: chronic disruption of rhythm (CDR) induced vs. Normal light/dark cycle (NLD).	Sleep assessment (Biotelemetery transmitter implantation, EEG, CBT, LA, NREM, REM, WAKE).<br>Fecal microbiota (shotgun meta-genomics), Conjugated bile acids measurement (LC-MS).	Prebiotics: higher relative abundance of Parabacteroides & Clostridium; lower secondary bile acid & glycoursodeoxycholic acid; prevention of decrease in evenness after CDR; effect on beta-diversity; significant sped up core body temperature (CBT) bathy phase realignment after chronic diurnal rhythm disruption (CDR), but not locomotor activity (LA).<br>Pre-CDR fecal microbiome & bile acids predicted recovery: lower cholic acid with higher Ruminiclostridium 5 predicted faster CBT realignment; higher Ruminiclostridium 5 with lower taurocholic acid predicted better NREM sleep recovery.	Prebiotics GOS/PDX: potential in modulating the level of secondary bile acid, increasing & modulating gut microbiota, and improving sleep/wake rhythm to a new night and dark rhythm.
[56] Yao et al. 2021	Experimental study in hypnotized male mice with specific pathogen-free (no sample size mentioned)	Acidic part of alcohol extract from G. Lucid mycelia (GLAA) vs. Solvent control	To evaluate the effect of GLAA on sleep, gut microbiome, transcriptome & metabolome	Hypnotic effect assessment (PBIT).<br>Neurotransmitters (5-HT, GABA, NE, LPS, PGN).<br>Sleep assessment (EEG).<br>Untargeted Transcriptomes.<br>Fecal microbiota (16s rRNA, metabolites).	GLAA: potentiation of pentobarbital-induced sleep; decrease in sleep latency; increase in sleeping time (dose-dependent); increase in 5-HT (50 & 100 mg/kg); no difference in richness at 100 mg/kg.<br>Antibiotics: inhibition of sleep-promoting effect; increase in 5 HT; decrease in richness of gut microbiota.<br>Significant increase in expression of the gene involved in the serotonergic synapse pathway.	GLAA: potential to improve sleep latency & duration; modulation of gut composition; increase in expression of gene reported to decrease wakefulness and involved in serotonergic synapse pathway; deprivation of effects after antibiotics, emphasising the beneficial effect of GLAA on gut microbiota.
[19] Bowers et al. 2022	Experimental study in 23-day SD rats (N=96)	Prebiotics diet containing GOS & PDX(Polydextrose)	To evaluate the effect of prebiotics GOS & PDX on improving sleep in response to repeated sleep disruption & during recovery sleep.	Sleep assessment (Sleep disruption, NREM, REM, WAKE).<br>Stress assessment (Social defeat).<br>Cognitive function assessment (OLT).	Prebiotics: improvement in sleep in response to repeated sleep disruption & during recovery sleep (increases in NREM & REM over time); increase in REM & NREM during 5 days of sleep disruption; increase in total sleep time during 24 hr of recovery;	GOS/PDX prebiotics: improvement of sleep quality in chronic sleep disruption.Parabateroides distanosis: positive correlation with improvement in sleep recovery.

				Fecal microbiota (16s rRNA), metabolites (LC/MS).	no change in NREM delta power; increase in relative abundance of *Parabacteroides distasonis.*	
[25] Chelliah et al 2022	Experimental study in C. Elegant & 5-wk ICR mice (no sample size mentioned)	Prebiotics FOS -> 1-ketose (GF-2) in Banana-peel oligosaccharide enzyme extract (BPOE) from Musa paradisiaca (banana peel)	To evaluate the efficacy & safety of the prebiotic GF-2 extract on the anti-stress effect.	Stress & anxiety assessment (EPM, OFT).<br>Cognitive function assessment (NOR).<br>Hormones/Neurotransmitters (5-HT, Corticosterone).<br>Fecal microbiota (16S rRNA).	Prebiotic GF-2: significant decrease in anxiety & stress; decrease in corticosterone; increase in relative abundance of *Oscilioapira*, *Mucisprillum & Parabateroides*; no cytotoxicity; anxiolytic effect.	BPOE & GF-2 extract: confirmed efficacy in probiotics growth & safety in C. Elegant.<br>GF-1: anxiolytic effect with behavior change & microbiota modulation.
[67] Li et al 2023	Experimental study in 6-wk Wistar rats (N=18)	Melatonin	To evaluate the effectiveness of melatonin intervention on neuropsychiatric behaviour.	Stress & anxiety assessment (OFT, EPM).<br>Depress assessment (SPT, FST).<br>Cognitive function assessment (MWM).<br>Fecal microbiota (16s rRNA), SCFAs (GC), bile acid (HPLC).<br>Pro-inflammatory cytokines (TNF-α, IL-1β).<br>Hormones/Neurotransmitters (Corticosteroid; Dopamine)	Sleep-deprived supplement with Melatonin (SDM): significantly lower corticosteroid; no difference in OFT; difference in EPM & SDM; prolongation of time in open arm; significantly higher total SCFA.<br>Melatonin: significant suppression of some pathogenic bacteria; decrease in F/B ratio.	Melatonin: reduction of anxiety behavior after exposure to chronic sleep deprivation; modulation of gut microbiota composition; preventive effect on SD-induced dysbiosis; suppression of secondary bile acid; maintenance of SCFAs in mice.
[34] Li et al 2023	Experimental study in 8-wk male ICR mice (N=90)	Melatonin	To evaluate the effectiveness of melatonin intervention on neuropsychiatric behaviour.	Multi-Modal Physiological Monitoring (MMPM).<br>Fecal microbiota (16s rRNA).	Controlled (CON) & sleep-restricted (SR) groups: time-varying oscillation in microbiota.<br>SR group: no circadian rhythm.<br>Melatonin: oscillation of gut microbiota and regain of circadian rhythm.	SR: alteration of colonic microbiomes by circadian rhythm loss, abolished after 24 hr.<br>Melatonin: promotion of restoration of circadian rhythm & GM composition, with a circadian trend similar to that of the control group.
[57] Xia et al 2023	Experimental study in 5-6 wk female BALB/c 4T1-tumor-bearing mice (N=40)	Tryptophan-rich whey protein isolation (WPI)	To investigate the effect of WPI on depression-like behaviour, GM & Tryp metabolism.	Stress & anxiety assessment (OFT, EPM).<br>Depression assessment (SPT, TST).	WPI & Fluoxetine (FLX): No significant difference among OFT, EPM, SPT &TST scores; significant increase in gut microbiota richness; promotion of evenness compared with tumour group.<br>Significant diversity in WPI between control vs. FLX.	WPI: decrease in depression-like behaviour in breast tumour mouse model; alteration of gut composition; promotion of integrity; maintenance of 5-HT production, potential treatment for tumour-related psychiatric symptoms.

				Neurotransmitters (LNAA, Tryptophan, 5-HT, 5-HTTP, 5-hydroxytryptophan, 5-HIAA).<br>Fecal microbiota (16s rRNA).		
[61] Cheng J, 2024	Experimental study C57BL/6 female mice (N= 20)	Probiotic fermented germinated grain complex (KFY): Inoculates Kombucha, Acetobacter, Saccharomycescerevisiae & Lactobacillus planetarium into a microbial fermentation medium composed of jujube, grains sprouts, grape seeds).	To evaluate the effect of KFY on sleep improvement &insomnia alleviation.	Sleep assessment: Pentobarbital sodium sleep synergy experiment (PCPA).<br>Emotional behavior assessment: OFT; grooming frequency.<br>Neurotransmitters: 5-HT; GABA.<br>Inflammatory biomarkers: IL-6; IL-1β; TNF-α.<br>Fecal sample: 16s rRNA gene sequencing;	KFY: significant increase in sleep duration time; reduction of insomnia induced anxiety behaviors (increased time at the central grid and increased grooming frequency; sustained 5-HT level & restoration of GABA levelafter induced insomnia with PCPA; significant reduction in IL-6.	KFY: prolongation of sleep duration & alleviation of anxiety behavior due to insomnia; restoration of neurotransmitters & gut microbiome composition.
[31] Wang L, 2024	Experimental study SPF grade male rats (N=36)	Banxia -Yiyiren (BY)	To investigate the effect of Banxia-Yiyoren in sleep regulatory snd its mechanism via gut-microbiota & metabolites.	Sleep assessment: Pentobarbital sodium sleep synergy experiment.<br>Emotional behavior assessment: EPM<br>Fecal sample: 16s rRNA gene sequencing; metabolites	BY (all dose levels): significant prolongation of time at the central area; gradual restoration of the microbes composition.<br>Insomnia: modulation of gut microbiota composition	Banxia-Yiyiren: activity on gut microbiota of PCPA-induced insomnia models rats and improving sleep and anxiety behavior induced by insomnia.
[55]<br>Li et al 2024	Experimental study in male C57BL/61 mice (N=70).<br>Dose-response analysis.	Citrus aurantium L. extract (FEMC)	To investigate the effect of Citrus aurantium L. extract on the anti-depressant effect in mice exposed to chronic unpredictable mild stress (CUMS).	Stress & anxiety assessment: (OFT, NSF).<br>Depression assessment (SPT).<br>Pro-inflammatory cytokines: (IL-6; TNF-α).<br>Fecal microbiota (16s rRNA).	FEMC: significant increase in sucrose preference; decrease in feeding latency; decrease in pro-inflammation level; significant reversal of reduced expression of gene involved in synaptic health; change in phylum level of gut flora composition induced by CUMS.	FEMC: improvement of depressive & anxiety caused by CUMS; modulation of gut composition changed by CUMS.
[59]<br>Zhang et al 2024	Experimental study in 5-7 wk wild-type C57BL/6L mice (N=30)	Limosilactobacillus reuteri	To identify a strain specific to Lm. reuteri (WLR01, vs. WLR06 vs. WLR08) and its effect on the alleviation of anxiety.	Stress & anxiety assessment (EPM & Y-maze).<br>Visceral sensitivity (CDR).<br>Fecal microbiota (16s rRNA).	Lm. Reuteri: prolongation of time in open arm EPM test (strain WLR01); regulation of gut microbiota structure (WLR01) by reversing the decrease in gut diversity by SD & restoring relative abundance of Lactobacillus (the effect maintains after WAS).	Lm. Reuteri (WLR01 strain): notable alleviation of anxiety-like behaviour; improvement of cognitive function.

				Pro-inflammatory cytokines (IL-6, TNF-α, IL-1β).<br>Hormones (CRH, CORT, MDA).		
[32]<br>Li et al 2024	Experimental study in 8-wk male ICR mice (N=24)	Eucalyptus essential oil (EEO)	To evaluate the effectiveness of EEO in treating sleep disorders.	Stress & anxiety assessment (OFT).<br>Hypnotic effectiveness (PBIT).<br>Neurotransmitters (GABA, glutamine, glycine, tryptophan, 5-HIAA).<br>Fecal microbiota (16s rRNA).	EEO: significant reduction of OFT speed & distance; shorter time in sleep latency; increase in sleep duration time; significant increase in brain GABA, glutamine, glycine, tryptophan, 5-HIAA & N-acetylserotonin; significant increase in alpha and beta-diversity; significant increase in relative abundance of Firmicutes, Proteobacteria & Actinobacteria.	EEO: improvement of sleep quantity; increase in brain level of sleep-promoting neurotransmitters; decrease in anxiety behaviour; modulation of gut microbiota composition; promotion of growth of probiotic group.
[68]<br>Tung et al 2024	Experimental study in 6-wk C5781/6J mice (N=18)	Black soy bean coat crude extract (BSSC)	To evaluate the efficacy of BSSC on immune regulation, gut microbiota & brain function in sleep-deprived mice.	Stress & anxiety assessment (OFT, EPM).<br>Cognitive function & memory (NORT, TM).<br>Fecal microbiota (16s rRNA, SCFAs).	BSSC: significant decrease in inflammation marker eosinophils & Th1; significant increase in total SCFAs; decrease in expression of circadian gene Per1 & Per2.	Potential of BSSC crude extract on issues associated with sleep deprivation.<br>Despite no improvement in anxiety, stress & memory capacity, restoration of gut homeostasis, decrease in inflammatory markers, and improvement of sleep deprivation by addressing dysregulation of circadian genes in mice.
[58]<br>Fan et al 2025	Experimental study in male mice (N=56)	GABA-enriched yogurt	To investigate the effect of GABA-enriched yogurt on mood, anxiety, cognitive memory & gut microbiota.	Stress & anxiety (OFT).<br>Memory test (Y-maze).<br>Fecal microbiota (16s rRNA).<br>Neurotransmitters (5-HT, Glu, GABA).<br>Oxidative stress (T-SOD).	GABA-enriched yogurt: prolongation of time in the central area of OFT; higher GABA in serum & brain; largest number of arm entries with a high score of spontaneous alternation behaviour (high dose); significant increase in relative abundance of Proteobacteria; lower in Firmicutes; higher F/B ratio.	GABA-rich yogurt: impact on mood, anxiety & cognitive memory caused by SD; improvement of gut microbiota structure resulting in a reduction in F/B ratio & an increase in probiotic family.
[33]<br>Huang et al 2025	Experimental study in 6-wk SPF-grade male ICR mice (N=40)	Moringa oleifera leaves (FM)	To investigate the effect of Moringa oleifera leaves on sleep improvement via gut-brain modulation.	Hypnotic effectiveness (PBIT).<br>Neurotransmitter (5-HT, GABA, Glu).<br>Brain tissue metabolic analysis (LC-MS).<br>Fecal microbiota (16s rRNA).	FM: shortening sleep latency; promotion of sleep duration (high dose); increase in brain GABA; decrease in glutamate and glutamate/GABA ratio; significant difference in gut microbiota; significant decrease in the Firmicutes group (high dose).	Fermented Moringa oleifera extract: sleep-promoting effect; increase in GABA & balance GABA/GLU ratio; modulation of gut microbiota structure & reduction of pathogenic species.
[60] Li P, 2025	Experimental study ICR SPF-grade mice (N=48)	Mixed probiotics Lactobacillales derived from traditional Xizang dairy product (Lactobacillus	To investigate the effect of mixed probiotic lactobacillales in insomnia alleviation.	Sleep assessment: Pentobarbital sodium sleep synergy experiment.<br>Emotional behavior assessment: OFT; EPM	Mixed probiotis & Diazepam: decrease in sleep latency; increase in total sleep duration; no change in anxiety like behavior; less shift in F/B ratio in insomnia.	Mixed probiotics: significant improvement of sleep quality; restoration of sleep related neurotransmitters; alteration of gut microbiome composition.

		helveeticus L551, Lacticaseibacillus paracasei L578 & Streptococcus thermophiles S709 group P)		Neurotransmitters: 5-HT; GABA; Glu; DA.<br>Fecal sample: 16s rRNA gene sequencing.	Probiotics: increase in 5-HT/TRP ratio; significant difference in beta diversity; enhancement of relative abundant of probiotic like Akkermasia & Acinetobacter.	
[69]<br>Sun et al 2025	Experimental study in 4-wk SPF mice (N=24)	Pediococcus acidilactici CCFM1344 (High-lactic acid producing strain) vs. FSDLZ42M3 (Low-lactic acid producing strain)	To investigate the effect of Pediococcus acidilactici CCFM1344 on stress-induced depression by comparing the impact between two strains (CCFM1344 vs. FSDLZ42M3)	Stress & anxiety assessment (OFT, EPM).<br>Depression assessment (TST).<br>Fecal microbiota (16s rRNA).<br>Un-targeted metabolites (LC-MS).	CCFM1344: no significant alteration of gut composition after exposure to stress; significant reversal of all; normal CPS level (increase in LPS level).	P. Acidilactici CCFM1344: potential alleviating depression & stress symptoms; alteration of some gut microbiota via increasing relative abundance of SCFA-producing genera and alleviation of intestinal barrier damage.
[70]<br>Zhang et al 2025	Experimental study in 5-wk female mice	Bifidobacterium animalis subsp. Lactis 3 isolated strains (WLBA3, WLBA6, WLBA7),	To investigate the effects of 3 different strains of Bifidobacterium animalis on inflammation & gut microbiota modulation.	Stress &anxiety assessment (EPM). Memory test (YM). Pro-inflammatory cytokines (L-1β, TNF-α, IL-6).<br>Hormones (MDA, CORT, CRH)<br>Fecal microbiota (16s rRNA).	WLBA3: most potent antioxidant effects; effective suppression of some inflammation markers; restoration of spontaneous alteration; restoration of richness & Firmicutes.<br>WLBA3 & WLBA7: significant increase in time spent on the rotarod test.<br>WLBA3 & WLBA6: significant increase in time in the open arm.	WLBA33 strain supplement in the mouse sleep-deprived model: potential to improve anxiety, cognitive memory & suppress inflammation; modulation of gut commotion & maintenance of homeostasis.
[30] Jia F, 2025	Experimental study in ICR SPF-grade mice (N=48)	Lily-Ziziphi Spinosae Semem decoction (LZ)	To investigate the effect of LZ in sleep promoting effect.	Sleep assessment: Pentobarbital sodium sleep synergy experiment; sleep/wake duration observation; Reversal reflex.<br>Inflammatory biomarkers: IL-6; IL-1β; TNF-α.<br>Neurotransmitters: 5-HT; GABA; Dopamine(DA); Norepinephrine (NE); Glutamate (Glu).<br>Fecal sample: 16s rRNA gene sequencing.	LZ: higher incidence of sleep with high dose; longer sleep duration & shorter sleep latency in medium & high dose; significant increase in hippocampus 5-HT & GABA; significant decrease in DA & Glu; increase in TNF-α .<br>No change in beta diversity between group, but promotion of growth of probioticthat involve with metabolism of carbohydrates & vitamins and inhibit growth of some opportunistic pathogens in high dose group.	LZ: sleep-improving effect, up-regulation of the abundance of beneficial bacteria & down-regulation of the pathogenic microbes; restoration of sleep promotion neurotransmitters & inflammatory biomarkers.

[29] Fang H., 2026	Experimental study in SD SPF-grade male rats (N=48)	Suanzaoren tang (SZRT) traditional Chinese medicine herbs	To investigate the effect of SZRT in exerting synergistic sleep promoting effect.	Sleep assessment: Pentobarbital sodium sleep synergy experiment.<br>Emotional behavior assessment: OFT.<br>Neurotransmitters: 5-HT; GABA; Dopamine(DA); Norepinephrine (NE); Glutamate (Glu).<br>Inflammatory biomarkers: IL-6; IL-1β; TNF-α.<br>Immunofluorescence: glial fibrillary acidic protein (GFAP); ionized calcium-binding adapter molecule (Iba-1); 5-HT1A; 5-HT2A receptors.<br>Fecal sample: 16s rRNA gene sequencing.	SZRT (medium & high dose): increase in total distance, number of grid crossing & time at the central area; restoration of IL-1β; significant decrease in IL-6.<br>SZRT (all doses): restoration of GABA, 5-HT & DA levels; decrease in NE & Glu levels; increase in expression of GFAP and Iba-1 levels; significant restoration of 5-HT1A & 5-HT2A.	SZRT: effective alleviation of PCPA-induced insomnia-like symptoms by restoration of vital neurotransmitters, gut microbiome composition & alleviation of anxiety-like behavior induced by insomnia; restoration of gut microbiome composition & improvement of dysbiosis.

Supplementary Table S3A Human Intervention Studies (28 Studies).

Framework: Adapted from Cochrane RoB 2.0 and ROBINS-I This table evaluates all interventional studies (RCTs, single-arm, and open-label trials).

Scoring Legend

[++] Low Risk: Robust methods explicitly reported (e.g., double-blind RCT, purely objective lab measures).
[+] Probably Low Risk: Generally sound design, but missing explicit methodological details in the summary.
[-] Probably High Risk: Inherent design flaws for this domain (e.g., lack of blinding combined with subjective questionnaires).
[--] High Risk: Definite bias risk (e.g., single-arm, open-label, non-randomized).
[?] Unclear / Unknown: Information not available in the provided summary (requires full-text review).

Reference	Study Design	Selection Bias (Randomization)	Performance Bias (Blinding)	Detection Bias (Outcome Measurement)	Attrition Bias (Drop-outs)	Selective Reporting	Overall Est. Risk
[63]	DB, Placebo-controlled RCT	+	++	++	?	+	**Low**
[45]	Single-arm, 12-wk	--	--	-	?	+	**High**
[22]	RCT, Placebo-controlled	+	+ *(DB implied)*	++	?	+	**Low**
[64]	RCT, cross-over	+	- *(Blind not stated)*	++	?	+	**Moderate**
[39]	DB RCT	+	++	++	?	+	**Low**
[52]	Single-arm, open-label	--	--	-	?	+	**High**
[43]	DB RCT, cross-over	+	++	++	?	+	**Low**
[41]	Pilot, non-randomized	--	--	-	?	+	**High**
[36]	Triple-blind, Placebo RCT	+	++	++	?	+	**Low**
[37]	RCT, cross-over	+	- *(Blind not stated)*	++	?	+	**Moderate**

Reference	Study Design	Selection Bias (Randomization)	Performance Bias (Blinding)	Detection Bias (Outcome Measurement)	Attrition Bias (Drop-outs)	Selective Reporting	Overall Est. Risk
[51]	DB, Placebo-controlled RCT	+	++	++	?	+	**Low**
[65]	Triple-blind, Placebo RCT	+	++	++	?	+	**Low**
[17]	Triple-blind, Placebo RCT	+	++	++	?	+	**Low**
[40]	DB, Placebo-controlled RCT	+	++	++	?	+	**Low**
[15]	DB, Placebo-controlled RCT	+	++	++	?	+	**Low**
[35]	RCT, cross-over vs placebo	+	+ *(Blind implied)*	++	?	+	**Moderate**
[50]	DB, Placebo RCT, cross-over	+	++	++	?	+	**Low**
[18]	DB, Placebo-controlled RCT	+	++	++	?	+	**Low**
[24]	Interventional (TCM vs TCM)	+	- *(No blind stated)*	++	?	+	**Moderate**
[20]	Non-randomized, open-label	--	--	-	?	+	**High**
[21]	DB, Placebo-controlled RCT	+	++	++	?	+	**Low**
[16]	RCT, Placebo-controlled	+	+ *(Blind implied)*	++	?	+	**Moderate**
[38]	DB RCT	+	++	++	?	+	**Low**

Reference	Study Design	Selection Bias (Randomization)	Performance Bias (Blinding)	Detection Bias (Outcome Measurement)	Attrition Bias (Drop-outs)	Selective Reporting	Overall Est. Risk
[27]	DB, Placebo-controlled RCT	+	++	++	?	+	**Low**
[44]	DB, Controlled RCT	+	++	++	?	+	**Low**
[48]	RCT	+	**-** *(No blind stated)*	++	?	+	**Moderate**
[13]	RCT, open-label, cross-over	+	--	**-** *(Subj. QOL)*	?	+	**High**
[28]	RCT, single-blind	+	-	**-** *(Subj. Mood)*	?	+	**Moderate**

Note: In trials lacking blinding (open-label, single-blind), "Detection Bias" is scored lower [-] because primary subjective outcomes (like the PSQI for sleep or DASS-21 for mood) are highly vulnerable to participant bias when they know what treatment they are receiving.

Supplementary Table S3B Human Observational/Cross-Sectional Studies (5 Studies).

Framework: Adapted from Cochrane ROBINS-I (Non-randomized) These studies assess populations at a single point in time, heavily relying on dietary recall.

Scoring Legend

[++] Low Risk: Robust methods explicitly reported (e.g., double-blind RCT, purely objective lab measures).
[+] Probably Low Risk: Generally sound design, but missing explicit methodological details in the summary.
[-] Probably High Risk: Inherent design flaws for this domain (e.g., lack of blinding combined with subjective questionnaires).
[--] High Risk: Definite bias risk (e.g., single-arm, open-label, non-randomized).
[?] Unclear / Unknown: Information not available in the provided summary (requires full-text review).

**Reference**	**Study Design**	**Confounding Bias (Adjustment for variables)**	**Selection Bias (Cohort Selection)**	**Exposure Bias (Diet/Habit Recall)**	**Detection Bias (Objective/Biomarker)**	**Selective Reporting**	**Overall Est. Risk**
[23]	Cross-sectional	**?** *(Not stated)*	**+**	**-**	++ (16S rRNA)	**+**	**Moderate**
[47]	Cross-sectional	**?** *(Not stated)*	**+**	**-**	++ (16S rRNA)	**+**	**Moderate**
[46]	Cross-sectional	**?** *(Not stated)*	**+**	**-**	++ (16S rRNA)	**+**	**Moderate**
[42]	Cross-sectional	**?** *(Not stated)*	**+**	**-**	++ (16s rRNA)	**+**	**Moderate**
[49]	Cross-sectional	**?** *(Not stated)*	**+**	**-**	++ (16S rRNA)	**+**	**Moderate**

Note: "Exposure Bias" is scored as [-] for all cross-sectional studies because they rely on self-reported dietary recalls or nutritional indexes (PAD, MNQI, DI-GM), which carry inherent, unavoidable human recall bias.

Supplementary Table S4 Animal (*In Vivo*) Studies (23 Studies).

Framework: Adapted from SYRCLE's Risk of Bias tool for animal experiments Animal studies notoriously suffer from poor reporting of randomization and caregiver blinding in their abstracts. However, they excel in objective detection methods. I have split "Detection Bias" into Objective (Microbiome/EEG) and Subjective (Behavioral tasks like the Open Field Test or Plus Maze).

Scoring Legend

[++] Low Risk: Robust methods explicitly reported (e.g., double-blind RCT, purely objective lab measures).
[+] Probably Low Risk: Generally sound design, but missing explicit methodological details in the summary.
[-] Probably High Risk: Inherent design flaws for this domain (e.g., lack of blinding combined with subjective questionnaires).
[--] High Risk: Definite bias risk (e.g., single-arm, open-label, non-randomized).
[?] Unclear / Unknown: Information not available in the provided summary (requires full-text review).

Reference	Animal Model	Selection Bias (Seq. Gen/Baseline)	Performance Bias (Caregiver Blinding)	Detection Bias (Objective: 16S, EEG, LC-MS)	Detection Bias (Subjective: Behavioral Tests)	Attrition Bias	Selective Reporting
[53]	F344 rats	?	?	++	*N/A*	?	+
[54]	F334 rats	?	?	++	*N/A*	?	+
[66]	ICR mice	?	?	++	- (OFT, EPM)	?	+
[26]	SD rats	?	?	++	*N/A*	?	+
[56]	Mice (SPF)	?	?	++	*N/A*	?	+
[19]	SD rats	?	?	++	- (OLT)	?	+
[25]	C.Elegan/ICR	?	?	++	- (EPM, OFT, NOR)	?	+
[67]	Wistar rats	?	?	++	- (OFT, SPT, MWM)	?	+
[34]	ICR mice	?	?	++	*N/A*	?	+
[57]	BALB/c mice	?	?	++	- (OFT, EPM, SPT)	?	+
[61]	C57BL/6 mice	?	?	++	- (OFT)	?	+
[31]	Rats (SPF)	?	?	++	- (EPM)	?	+

Reference	Animal Model	Selection Bias (Seq. Gen/Baseline)	Performance Bias (Caregiver Blinding)	Detection Bias (Objective: 16S, EEG, LC-MS)	Detection Bias (Subjective: Behavioral Tests)	Attrition Bias	Selective Reporting
[55]	C57BL/61 mice	?	?	++	- (OFT, NSF, SPT)	?	+
[59]	C57BL/6L mice	?	?	++	- (EPM, Y-maze)	?	+
[32]	ICR mice	?	?	++	- (OFT)	?	+
[68]	C5781/6J mice	?	?	++	- (OFT, EPM, TM)	?	+
[58]	Male mice	?	?	++	- (OFT, Y-maze)	?	+
[33]	ICR mice (SPF)	?	?	++	*N/A*	?	+
[60]	ICR mice (SPF)	?	?	++	- (OFT, EPM)	?	+
[69]	SPF mice	?	?	++	- (OFT, EPM, TST)	?	+
[70]	Female mice	?	?	++	- (EPM, YM)	?	+
[30]	ICR mice (SPF)	?	?	++	- (Reversal reflex)	?	+
[29]	SD rats (SPF)	?	?	++	- (OFT)	?	+

Note: Unless an animal study explicitly states that the observer grading the behavioral tests (OFT, EPM, SPT, etc.) was blinded to the animal's diet group, it carries a high risk [-] of subjective detection bias. However, objective machine-read data (16s rRNA, LC-MS, HPLC, EEG) completely bypasses human observer bias [++].

References

1. Morin, C. M.; Jarrin, D. C., Epidemiology of Insomnia: Prevalence, Course, Risk Factors, and Public Health Burden. Sleep Medicine Clinics 2022, 17, (2), 173-191. https://doi.org/10.1016/j.jsmc.2022.03.003.
2. Cryan, J.F.; Dinan, T.G. Mind-altering microorganisms: The impact of the gut microbiota on brain and behavior. *Nat. Rev. Neurosci.* **2012**, *13*, 701–712. https://doi.org/10.1038/nrn3346.
3. Silva, Y. P.; Bernardi, A.; Frozza, R. L., The Role of Short-Chain Fatty Acids From Gut Microbiota in Gut-Brain Communication. Front Endocrinol (Lausanne) 2020, 11, 25. https://doi.org/10.3389/fendo.2020.00025.
4. Vargas, I.; Perlis, M. L., Insomnia and depression: clinical associations and possible mechanistic links. Curr Opin Psychol 2020, 34, 95-99. https://doi.org/10.1016/j.copsyc.2019.11.004.
5. Dalile, B.; Van Oudenhove, L.; Vervliet, B.; Verbeke, K., The role of short-chain fatty acids in microbiota–gut–brain communication. Nature Reviews Gastroenterology & Hepatology 2019, 16, (8), 461-478.https://doi.org/10.1038/s41575-019-0157-3.
6. Mediavilla, C., Bidirectional gut-brain communication: A role for orexin-A. Neurochem Int 2020, 141, 104882. https://doi.org/10.1016/j.neuint.2020.104882.

7. Morrison, D.J.; Preston, T. Formation of short chain fatty acids by the gut microbiota and their health effects. *Curr. Nutr. Rep.* **2016**, *5*, 58–67. https://doi.org/10.1007/s13668-016-0172-0.
8. Khan, M.A.; Tofighi, S. Orexin and Its Role in Sleep and Wakefulness: A Review. *J. Sleep Res.* **2022**, *31*, e13550. https://doi.org/10.1016/j.peptides.2023.171007.
9. Riemann, D.; Nissen, C.; Perlis, M.L. The neurobiology, diagnosis, and treatment of chronic insomnia. *Lancet Psychiatry* **2017**, *4*, 486–500. https://doi.org/10.1016/S2215-0366(17)30114-3.
10. Tung, C.-L.; Wu, J.-H.; Chang, H.-C.; Xu, J.-W.; Yang, Y.-C. S. H.; Wu, C. W.; Tung, Y.-T., Effects of black soybean seed coat (BSSC) crude extract on the immune regulation, gut microbiota, and brain function of mice with sleep deprivation. Journal of Functional Foods 2024, 119, 106335. https://doi.org/10.1016/j.jff.2024.106335
11. Sudo, N.; Chida, Y.; Aiba, Y.; Sonoda, J.; Oyama, N.; Kubo, C. Postnatal microbial colonization programs the hypothalamic-pituitary-adrenal system for stress regulation. *J. Physiol.* **2004**, *558*, 263–275. https://doi.org/10.1113/jphysiol.2004.063388.
12. Chen, S.; Li, H.; Ning, M.; Yu, B. Y. M.; Wu, S.; Cheng, W. Y.; Li, Y.; Yeung, W. F., The association between gut microbiota and insomnia: A systematic review and meta-analysis. Sleep Med Rev 2026, 86, 102236. https://doi.org/10.1016/j.smrv.2026.102236.
13. Robinson, L. A.; Lennon, S.; Pegel, A. R.; Strickland, K. P.; Feeley, C. A.; Watts, S. O.; W, J. V. D. P.; Roberts, M. D.; Greene, M. W.; Frugé, A. D., A Randomized Controlled Crossover Lifestyle Intervention to Improve Metabolic and Mental Health in Female Healthcare Night-Shift Workers. Nutrients 2025, 17, (21).https://doi.org/10.3390/nu17213342.
14. Page, M.J.; McKenzie, J.E.; Bossuyt, P.M.; Boutron, I.; Hoffmann, T.C.; Mulrow, C.D.; Shamseer, L.; Tetzlaff, J.M.; Akl, E.A.; Brennan, S.E.; et al. The PRISMA 2020 statement: An updated guideline for reporting systematic reviews. *Syst. Rev.* **2021**, *10*, 89. https://doi.org/10.1186/s13643-021-01624-y.
15. Tian, P.; Lan, Y.; Jin, Z.; Hang, F.; Mao, X.; Jin, X.; Wang, G.; Chen, W. Regulation of sleep and circadian rhythms by S-adenosylmethionine-producing probiotics. *Engineering* **2026**, *57*, 250–261. https://doi.org/10.1016/j.eng.2024.12.025.
16. Wang, J.; Liu, Y.; Zhu, C.; Wang, Z.; Wang, S.; Fang, S.; Xu, F. Gut microbiota and immune regulation by *Lactobacillus delbrueckii* subsp. bulgaricus LB42: From preclinical safety assessment to clinical evidence. *Food Chem. Toxicol.* **2026**, *207*, 115851. https://doi.org/10.1016/j.fct.2025.115851.
17. Li, J.; Zhao, J.; Ze, X.; Li, L.; Li, Y.; Zhou, Z.; Wu, S.; Jia, W.; Liu, M.; Li, Y.; et al. *Lacticaseibacillus paracasei* 207-27 alters the microbiota-gut-brain axis to improve wearable device-measured sleep duration in healthy adults: A randomized, double-blind, placebo-controlled trial. *Food Funct.* **2024**, *15*, 10732–10745. https://doi.org/10.1039/d4fo01684j.
18. Tanihiro, R.; Yuki, M.; Sasai, M.; Haseda, A.; Kagami-Katsuyama, H.; Hirota, T.; Honma, N.; Nishihira, J., Effects of Prebiotic Yeast Mannan on Gut Health and Sleep Quality in Healthy Adults: A Randomized, Double-Blind, Placebo-Controlled Study. Nutrients 2024, 16, (1). https://doi.org/10.3390/nu16010141.
19. Bowers, S.J.; Summa, K.C.; Thompson, R.S.; Gonzalez, A.; Vargas, F.; Olker, C.; Jiang, P.; Lowry, C.A.; Dorrestein, P.C.; Knight, R.; et al. A prebiotic diet alters the fecal microbiome and improves sleep in response to sleep disruption in rats. *Front. Neurosci.* **2022**, *16*, 889211. https://doi.org/10.3389/fnins.2022.889211.
20. Lau, R.I.; Su, Q.; Ching, J.Y.L.; Lui, R.N.; Chan, T.T.; Wong, M.T.L.; Lau, L.H.S.; Wing, Y.K.; Chan, R.N.Y.; Kwok, H.Y.H.; et al. Fecal microbiota transplantation for sleep disturbance in post-acute COVID-19 syndrome. *Clin. Gastroenterol. Hepatol.* **2024**, *22*, 2487–2496 e2486. https://doi.org/10.1016/j.cgh.2024.06.004.
21. Guan, Y.; Zhu, R.; Zhao, W.; Wang, L.; You, L.; Zeng, Z.; Jiang, Q.; Zhu, Z.; Gou, J.; Zhang, Q.; et al. Effects of *Lacticaseibacillus paracasei* K56 on perceived stress among pregraduate students: A double-blind, randomized, placebo-controlled trial. *Front. Nutr.* **2025**, *12*, 1544713. https://doi.org/10.3389/fnut.2025.1544713.
22. Valle, M.; Vieira, I.A.; Fino, L.C.; Gallina, D.A.; Esteves, A.M.; da Cunha, D.T.; Cabral, L.; Benatti, F.B.; Marostica Junior, M.R.; Batista, A.G.; et al. Immune status, well-being and gut microbiota in military supplemented with synbiotic ice cream and submitted to field training: A randomised clinical trial. *Br. J. Nutr.* **2021**, *126*, 1794–1808. https://doi.org/10.1017/S0007114521000568.

23. Meng, Y.; Ma, W.; Li, X.; Zhang, N., A Novel Dietary Index for Gut Microbiota (DI-GM) is Associated With Inflammation, Mental Health, and Tumor Biomarkers in Adults: A Cross-Sectional Study. Food Science and Nutrition 2025, 13, (10). https://doi.org/10.1002/fsn3.70951.
24. Zeng, H.; Xu, J.; Zheng, L.; Zhan, Z.; Fang, Z.; Li, Y.; Zhao, C.; Xiao, R.; Zheng, Z.; Li, Y.; Yang, L., Traditional Chinese herbal formulas modulate gut microbiome and improve insomnia in patients with distinct syndrome types: insights from an interventional clinical study. Frontiers in Cellular and Infection Microbiology 2024, 14.https://doi.org/10.3389/fcimb.2024.1395267.
25. Chelliah, R.; Park, S.J.; Oh, S.; Lee, E.; Daliri, E.B.-M.; Elahi, F.; Park, C.R.; Sultan, G.; Madar, I.H.; Oh, D.H. Unveiling the potentials of bioactive oligosaccharide1-kestose (GF2) from *Musa paradisiaca* Linn peel with an anxiolytic effect based on gut microbiota modulation in stressed mice model. *Food Biosci.* **2022**, *49*, 101881. https://doi.org/10.1016/j.fbio.2022.101881.
26. Thompson, R.S.; Gaffney, M.; Hopkins, S.; Kelley, T.; Gonzalez, A.; Bowers, S.J.; Vitaterna, M.H.; Turek, F.W.; Foxx, C.L.; Lowry, C.A.; et al. Ruminiclostridium 5, Parabacteroides distasonis, and bile acid profile are modulated by prebiotic diet and associate with facilitated sleep/clock realignment after chronic disruption of rhythms. *Brain Behav. Immun.* **2021**, *97*, 150–166. https://doi.org/10.1016/j.bbi.2021.07.006.
27. Arce-Lopez, B.; Bazan, G.X.; Molina, S.; Crespo, M.C.; Garcia-Beccaria, M.; Cruz-Gil, S.; Fernandez-Diaz, C.M.; Ramirez de Molina, A.; Ramos-Ruiz, R.; Espinosa-Salinas, M.I. Effect of fiber-modified kombucha tea on gut microbiota in healthy population: A randomized controlled trial (RCT). *Curr. Res. Food Sci.* **2025**, *11*, 101130. https://doi.org/10.1016/j.crfs.2025.101130.
28. Sasaki, H.; Masutomi, H.; Yamauchi, Y.; Ishihara, K.; Fukuda, S. Effectiveness of personalized granola tailored to the gut microbiota for improving gut environment and mood states. *Front. Microbiol.* **2025**, *16*, 1607918. https://doi.org/10.3389/fmicb.2025.1607918.
29. Fang, H.; Wang, Y. H.; Yang, L.; Che, Y. H.; Liu, F. L.; Liu, H., Revealing the mechanism of suanzaoren tang against insomnia via integrated metabolomics and gut microbiota analysis. Journal of Pharmaceutical and Biomedical Analysis 2026, 269. https://doi.org/10.1016/j.jpba.2025.117231.
30. Jia, F.; Zheng, H.; Xu, Y.; Jiang, J.; Wu, Y.; Liu, J.; He, K.; Yang, Y., Material basis and sleep-improving mechanisms of Lily-Ziziphi Spinosae Semen decoction: systematic evidence from LC-MS, network pharmacology and animal experiments. Food Science and Human Wellness 2025, 14, (9).https://doi.org/10.26599/FSHW.2025.9250493.
31. Wang, L.; Qi, X.; Wang, S.; Tian, C.; Zou, T.; Liu, Z.; Chen, Q.; Chen, Y.; Zhao, Y.; Li, S.; Yang, M.; Chai, N., Banxia-Yiyiren alleviates insomnia and anxiety by regulating the gut microbiota and metabolites of PCPA-induced insomnia model rats. Frontiers in Microbiology 2024, 15. https://doi.org/10.3389/fmicb.2024.1405566
32. Li, X.; Zhang, Y.; Zhang, Q.; Cao, A.; Feng, J. Eucalyptus essential oil exerted a sedative-hypnotic effect by influencing brain neurotransmitters and gut microbes via the gut microbiota-brain axis. *Front. Pharmacol.* **2024**, *15*, 1464654. https://doi.org/10.3389/fphar.2024.1464654.
33. Huang, S.; Wu, K.; Guo, Y.; Mu, H.; Sheng, J.; Tian, Y.; Liu, J.; Zhao, C., Integrated Approach Reveals Fermented Moringa oleifera Leaves Extracts' Impact on Mouse Sleep. Foods 2025, 14, (17). https://doi.org/10.3390/foods14172952.
34. Li, W.; Wang, Z.; Cao, J.; Dong, Y.; Chen, Y. Melatonin improves the homeostasis of mice gut microbiota rhythm caused by sleep restriction. *Microbes Infect.* **2023**, *25*, 105121. https://doi.org/10.1016/j.micinf.2023.105121.
35. Kortman, G.A.M.; Hester, E.R.; Schaafsma, A.; Mulder, J.; Mallee, L.; Nauta, A. Gut microbiome composition and functionality impact the responsiveness to a dairy-based product containing galacto-oligosaccharides for improving sleep quality in adults. *Benef. Microbes* **2024**, *15*, 373–385. https://doi.org/10.1163/18762891-bja00017.
36. Makela, S.M.; Griffin, S.M.; Reimari, J.; Evans, K.C.; Hibberd, A.A.; Yeung, N.; Ibarra, A.; Junnila, J.; Turunen, J.; Beboso, R.; et al. Efficacy and safety of *Lacticaseibacillus paracasei* Lpc-37(R) in students facing examination stress: A randomized, triple-blind, placebo-controlled clinical trial (the ChillEx study). *Brain Behav. Immun. Health* **2023**, *32*, 100673. https://doi.org/10.1016/j.bbih.2023.100673.

37. Mysonhimer, A.R.; Cannavale, C.N.; Bailey, M.A.; Khan, N.A.; Holscher, H.D. Prebiotic consumption alters microbiota but not biological markers of stress and inflammation or mental health symptoms in healthy adults: A randomized, controlled, crossover trial. *J. Nutr.* **2023**, *153*, 1283–1296. https://doi.org/10.1016/j.tjnut.2023.02.015.
38. Santamarina, A.B.; Filho, V.N.; de Freitas, J.A.; Franco, L.A.M.; Martins, R.C.; Fonseca, J.V.; Orellana Turri, J.A.; Hufnagel, M.T.; Demarque, D.P.; da Silva, B.; et al. Nutraceutical blends promote weight loss, inflammation reduction, and better sleep: The role of *Faecalibacterium prausnitzii* in overweight adults-a double-blind trial. *Mol. Nutr. Food Res.* **2025**, *69*, e202400806. https://doi.org/10.1002/mnfr.202400806.
39. Colombo, J.; Carlson, S.E.; Algarin, C.; Reyes, S.; Chichlowski, M.; Harris, C.L.; Wampler, J.L.; Peirano, P.; Berseth, C.L. Developmental effects on sleep-wake patterns in infants receiving a cow's milk-based infant formula with an added prebiotic blend: A Randomized Controlled Trial. *Pediatr. Res.* **2021**, *89*, 1222–1231. https://doi.org/10.1038/s41390-020-1044-x.
40. Lozar Krivec, J.; Bratina, P.; Valcl, A.; Lozar Manfreda, K.; Petrovcic, A.; Benedik, E.; Obermajer, T.; Bogovic Matijasic, B.; Setina, U.; Rupnik, M.; et al. Effects of *Limosilactobacillus* reuteri DSM 17938 in neonates exposed to antibiotics: A randomised controlled trial. *Benef. Microbes* **2024**, *16*, 157–169. https://doi.org/10.1163/18762891-bja00049.
41. Lawrence, K.; Myrissa, K.; Toribio-Mateas, M.; Minini, L.; Gregory, A.M. Trialling a microbiome-targeted dietary intervention in children with ADHD-the rationale and a non-randomised feasibility study. *Pilot Feasibility Stud.* **2022**, *8*, 108. https://doi.org/10.1186/s40814-022-01058-4.
42. Meng, Y.; Tian, J.; Xiu Li, X.; Xu, Z. Associations of MIND and DI-GM dietary scores with depression, anxiety, and gut microbiota in patients with colon cancer: A cross-sectional study. *Front. Nutr.* **2025**, *12*, 1655051. https://doi.org/10.3389/fnut.2025.1655051.
43. Baldi, S.; Pagliai, G.; Dinu, M.; Di Gloria, L.; Nannini, G.; Curini, L.; Pallecchi, M.; Russo, E.; Niccolai, E.; Danza, G.; et al. Effect of ancient Khorasan wheat on gut microbiota, inflammation, and short-chain fatty acid production in patients with fibromyalgia. *World J. Gastroenterol.* **2022**, *28*, 1965–1980. https://doi.org/10.3748/wjg.v28.i18.1965.
44. Inoue, R.; Suzuki, K.; Takaoka, M.; Narumi, M.; Naito, Y., Effects of Dietary Fiber Supplementation on Gut Microbiota and Bowel Function in Healthy Adults: A Randomized Controlled Trial. Microorganisms 2025, 13, (9). https://doi.org/10.3390/microorganisms13092068.
45. Butler, M. I.; Bastiaanssen, T. F. S.; Long-Smith, C.; Berding, K.; Morkl, S.; Cusack, A. M.; Strain, C.; Busca, K.; Porteous-Allen, P.; Claesson, M. J.; Stanton, C.; Cryan, J. F.; Allen, D.; Dinan, T. G., Recipe for a Healthy Gut: Intake of Unpasteurised Milk Is Associated with Increased Lactobacillus Abundance in the Human Gut Microbiome. Nutrients 2020, 12, (5). https://doi.org/10.3390/nu12051468.
46. Chen, Q.; Fan, R.; Song, L.; Wang, S.; You, M.; Cai, M.; Wu, Y.; Li, Y.; Xu, M. Association of methyl donor nutrients dietary intake and sleep disorders in the elderly revealed by the intestinal microbiome. *Food Funct.* **2024**, *15*, 6335–6346. https://doi.org/10.1039/d4fo01303d.
47. Jiang, X.; Wang, X.; Zhang, M.; Yu, L.; He, J.; Wu, S.; Yan, J.; Zheng, Y.; Zhou, Y.; Chen, Y. Associations between specific dietary patterns, gut microbiome composition, and incident subthreshold depression in Chinese young adults. *J. Adv. Res.* **2024**, *65*, 183–195. https://doi.org/10.1016/j.jare.2024.05.030.
48. Lane, M.M.; McGuinness, A.J.; Mohebbi, M.; Lotfaliany, M.; Loughman, A.; O'Hely, M.; O'Neil, A.; Batti, J.; Kotowicz, M.; Berk, M.; et al. Food- vs. supplement-based very-low-energy diets and gut microbiome composition in women with high body mass index: A randomized controlled trial. *Cell Rep. Med.* **2025**, *6*, 102417. https://doi.org/10.1016/j.xcrm.2025.102417.
49. Tao, Y.; Wu, M.; Su, B.; Lin, H.; Li, Q.; He, Y.; Zhong, T.; Xiao, Y.; Yu, X. Host-gut microbiota interactions: Exploring the potential role of vitamin B1 and B2 in the microbiota-gut-brain axis and anxiety, stress, and sleep quality. *Nutrients* **2025**, *17*, 1894. https://doi.org/10.3390/nu17111894.
50. Gillies, N.A.; Wilson, B.C.; Miller, J.R.; Roy, N.C.; Scholey, A.; Braakhuis, A.J. Effects of a flavonoid-rich blackcurrant beverage on markers of the gut-brain axis in healthy females: Secondary findings from a 4-week randomized crossover control trial. *Curr. Dev. Nutr.* **2024**, *8*, 102158. https://doi.org/10.1016/j.cdnut.2024.102158.

51. Crichton, M.; Marshall, S.; Marx, W.; Isenring, E.; Vazquez-Campos, X.; Dawson, S.L.; Lohning, A. Effect of ginger root powder on gastrointestinal bacteria composition, gastrointestinal symptoms, mental health, fatigue, and quality of life: A double-blind placebo-controlled trial. *J. Nutr.* **2023**, *153*, 3193–3206. https://doi.org/10.1016/j.tjnut.2023.09.002.
52. Kobayashi, A.; Nagashima, K.; Hu, A.; Harada, Y.; Kobayashi, H. Effectiveness and safety of kamikihito, a traditional Japanese medicine, in managing anxiety among female patients with intractable chronic constipation. *Complement. Ther. Clin. Pract.* **2022**, *46*, 101526. https://doi.org/10.1016/j.ctcp.2021.101526.
53. Thompson, R.S.; Roller, R.; Mika, A.; Greenwood, B.N.; Knight, R.; Chichlowski, M.; Berg, B.M.; Fleshner, M. Dietary prebiotics and bioactive milk fractions improve NREM sleep, enhance REM sleep rebound and attenuate the stress-induced decrease in diurnal temperature and gut microbial alpha diversity. *Front. Behav. Neurosci.* **2016**, *10*, 240. https://doi.org/10.3389/fnbeh.2016.00240.
54. Thompson, R.S.; Vargas, F.; Dorrestein, P.C.; Chichlowski, M.; Berg, B.M.; Fleshner, M. Dietary prebiotics alter novel microbial dependent fecal metabolites that improve sleep. *Sci. Rep.* **2020**, *10*, 3848. https://doi.org/10.1038/s41598-020-60679-y.
55. Li, T.; Zeng, G.; Zhu, L.; Wu, Y.; Zhang, Q.; Fu, F.; Su, D.; Li, G.; Li, Q.; Shan, Y. *Citrus aurantium* L. extract alleviate depression by inhibiting gut microbiota-mediated inflammation in mice. *Food Sci. Hum. Wellness* **2024**, *13*, 3403–3414. https://doi.org/10.26599/FSHW.2023.9250025.
56. Yao, C.; Wang, Z.; Jiang, H.; Yan, R.; Huang, Q.; Wang, Y.; Xie, H.; Zou, Y.; Yu, Y.; Lv, L. *Ganoderma lucidum* promotes sleep through a gut microbiota-dependent and serotonin-involved pathway in mice. *Sci. Rep.* **2021**, *11*, 13660. https://doi.org/10.1038/s41598-021-92913-6.
57. Xia, S.; Maitiniyazi, G.; Liu, Y.; Chen, Y.; Guo, M.; He, J.; Tao, W.; Li, Z. Whey protein isolate attenuates depression-like behavior developed in a mouse model of breast tumor. *Food Res. Int.* **2023**, *169*, 112849. https://doi.org/10.1016/j.foodres.2023.112849.
58. Fan, X.; Zhou, H.; Shen, Q.; Quan, W.; Shi, Z.; Wu, Z.; Chen, B.; Pan, D.; Luo, J. Gamma-aminobutyric acid-enriched yogurt alleviates anxiety and memory decline in mice with circadian rhythm disorders via the gut-brain axis. *Food Biosci.* **2025**, *63*, 105676. https://doi.org/10.1016/j.fbio.2024.105676.
59. Zhang, L.; Zhang, S.; Jiang, M.; Ni, X.; Du, M.; Jiang, H.; Bi, M.; Wang, Y.; Liu, C.; Liu, S., Limosilactobacillus reuteri Alleviates Anxiety-like Behavior and Intestinal Symptoms in Two Stressed Mouse Models. Nutrients 2024, 16, (18). https://doi.org/10.3390/nu16183209
60. Li, P.; Yang, L.; Shao, X.; Zou, Z.; Shi, H.; Sun, Y.; Wu, X.; Li, Z.; Li, Y.; Li, Z., Lactobacillales derived from traditional Xizang dairy products improve insomnia and restore neurotransmitter-metabolic profiles via gut microbiota in PCPA-induced mice. Microbiological Research 2025, 300. https://doi.org/10.1016/j.micres.2025.128276.
61. Cheng, J.; Wu, Q.; Sun, R.; Li, W.; Wang, Z.; Zhou, M.; Yang, T.; Wang, J.; Lyu, Y.; Yue, C., Protective effects of a probiotic-fermented germinated grain complex on neurotransmitters and sleep quality in sleep-deprived mice. Frontiers in Microbiology 2024, 15. https://doi.org/10.3389/fmicb.2024.1438928.
62. Mogavero, M.P.; Silvani, A.; Lanza, G.; DelRosso, L.M.; Ferini-Strambi, L.; Ferri, R. Targeting Orexin Receptors for the Treatment of Insomnia: From Physiological Mechanisms to Current Clinical Evidence and Recommendations. *Nat. Sci. Sleep* **2023**, *15*, 17–38. http://doi.org/10.2147/nss.s201994.
63. Sawada, D.; Kuwano, Y.; Tanaka, H.; Hara, S.; Uchiyama, Y.; Sugawara, T.; Fujiwara, S.; Rokutan, K.; Nishida, K. Daily intake of Lactobacillus gasseri CP2305 relieves fatigue and stress-related symptoms in male university Ekiden runners: A double-blind, randomized, and placebo-controlled clinical trial. *J. Funct. Foods* **2019**, *57*, 465–476, https://doi.org/10.1016/j.jff.2019.04.022.
64. Schaafsma, A.; Mallee, L.; Belt, M.v.D.; Floris, E.; Kortman, G.; Veldman, J.; Ende, D.v.D.; Kardinaal, A. The Effect of A Whey-Protein and Galacto-Oligosaccharides Based Product on Parameters of Sleep Quality, Stress, and Gut Microbiota in Apparently Healthy Adults with Moderate Sleep Disturbances: A Randomized Controlled Cross-Over Study. *Nutrients* **2021**, *13*, 2204, https://doi.org/10.3390/nu13072204.
65. Li, J.; Li, Y.; Zhao, J.; Li, L.; Wang, Y.; Chen, F.; Li, Y.; Cheng, R.; He, F.; Ze, X.; et al. Effects of Bifidobacterium breve 207-1 on regulating lifestyle behaviors and mental wellness in healthy adults based on the microbiome-gut-brain axis: a randomized, double-blind, placebo-controlled trial. *Eur. J. Nutr.* **2024**, *63*, 2567–2585, https://doi.org/10.1007/s00394-024-03447-2.

66. Yu, L.; Han, X.; Cen, S.; Duan, H.; Feng, S.; Xue, Y.; Tian, F.; Zhao, J.; Zhang, H.; Zhai, Q.; Chen, W., Beneficial effect of GABA-rich fermented milk on insomnia involving regulation of gut microbiota. Microbiol Res 2020, 233. https://doi.org/10.1016/j.micres.2020.126409
67. Li, B.; Hsieh, Y. R.; Lai, W. D.; Tung, T. H.; Chen, Y. X.; Yang, C. H.; Fang, Y. C.; Huang, S. Y., Melatonin Ameliorates Neuropsychiatric Behaviors, Gut Microbiome, and Microbiota-Derived Metabolites in Rats with Chronic Sleep Deprivation. Int J Mol Sci 2023, 24, (23). https://doi.org/10.3390/ijms242316820
68. Tung, C.-L.; Wu, J.-H.; Chang, H.-C.; Xu, J.-W.; Yang, Y.-C. S. H.; Wu, C. W.; Tung, Y.-T., Effects of black soybean seed coat (BSSC) crude extract on the immune regulation, gut microbiota, and brain function of mice with sleep deprivation. Journal of Functional Foods 2024, 119, 106335. https://doi.org/10.1016/j.jff.2024.106335
69. Sun, J.; Jin, X.; Yang, H.; Qian, X.; Tian, P.; Wang, G.; Zhao, J., Pediococcus acidilactici CCFM1344 alleviates chronic stress-induced depression by mitigating Gut microbiota imbalance and Neuroinflammation. Food Bioscience 2025, 63, 105701. https://doi.org/10.1016/j.fbio.2024.105701
70. Zhang, Y.-K.; Zhang, L.; Ni, X.; Zhang, S.-W.; Jiang, M.-Z.; Zhang, S.-L.; Xiao, G.-X.; Jiang, H.; Bi, M.-X.; Wang, Y.-L.; Liu, C.; Liu, S.-J., Bifidobacterium animalis subsp. lactis genome resources and metabolite profiling at the strain level and their ability to alleviate anxiety-like behavior in a sleep-deprived mouse model. Engineering Microbiology 2025, 5, (4), 100228. https://doi.org/10.1016/j.engmic.2025.100228

Disclaimer/Publisher's Note: The statements, opinions and data contained in all publications are solely those of the individual author(s) and contributor(s) and not of MDPI and/or the editor(s). MDPI and/or the editor(s) disclaim responsibility for any injury to people or property resulting from any ideas, methods, instructions or products referred to in the content.
